# A comparison framework and guideline of clustering methods for mass cytometry data

**DOI:** 10.1186/s13059-019-1917-7

**Published:** 2019-12-23

**Authors:** Xiao Liu, Weichen Song, Brandon Y. Wong, Ting Zhang, Shunying Yu, Guan Ning Lin, Xianting Ding

**Affiliations:** 1 State Key Laboratory of Oncogenes and Related Genes, Institute for Personalized Medicine, School of Biomedical Engineering, Shanghai Jiao Tong University, 1954 Huashan Road, Shanghai, 200030 China; 2 Shanghai Key Laboratory of Psychotic Disorders, Shanghai Mental Health Center, Shanghai Jiao Tong University School of Medicine, 600 South Wanping Road, Shanghai, 200030 China; 3 Department of Biomedical Engineering, Johns Hopkins University, Baltimore, MD 21218 USA

**Keywords:** Mass cytometry, CyTOF, Cell population, Clustering tools, Comparison

## Abstract

**Background:**

With the expanding applications of mass cytometry in medical research, a wide variety of clustering methods, both semi-supervised and unsupervised, have been developed for data analysis. Selecting the optimal clustering method can accelerate the identification of meaningful cell populations.

**Result:**

To address this issue, we compared three classes of performance measures, “precision” as external evaluation, “coherence” as internal evaluation, and stability, of nine methods based on six independent benchmark datasets. Seven unsupervised methods (Accense, Xshift, PhenoGraph, FlowSOM, flowMeans, DEPECHE, and kmeans) and two semi-supervised methods (Automated Cell-type Discovery and Classification and linear discriminant analysis (LDA)) are tested on six mass cytometry datasets. We compute and compare all defined performance measures against random subsampling, varying sample sizes, and the number of clusters for each method. LDA reproduces the manual labels most precisely but does not rank top in internal evaluation. PhenoGraph and FlowSOM perform better than other unsupervised tools in precision, coherence, and stability. PhenoGraph and Xshift are more robust when detecting refined sub-clusters, whereas DEPECHE and FlowSOM tend to group similar clusters into meta-clusters. The performances of PhenoGraph, Xshift, and flowMeans are impacted by increased sample size, but FlowSOM is relatively stable as sample size increases.

**Conclusion:**

All the evaluations including precision, coherence, stability, and clustering resolution should be taken into synthetic consideration when choosing an appropriate tool for cytometry data analysis. Thus, we provide decision guidelines based on these characteristics for the general reader to more easily choose the most suitable clustering tools.

## Background

During the last decade, single-cell technology has progressed tremendously. With the ability to simultaneously measure multiple features at the single-cell level, biologists are now capable of depicting biological and pathological processes with unprecedented complexity [1]. Mass cytometry, which is achieved with Cytometry by Time-Of-Flight (CyTOF), is an advanced experimental technology that measures levels of multiple proteins (up to 40) in a large amount (usually several millions) of cells [2]. The supreme ability to access a large panel of proteins simultaneously makes CyTOF useful in drug optimization [3], vaccine development [4], and disease marker discovery [5]. Compared to the well-known technology of single-cell RNA-sequencing (scRNA-seq) [6–8], which processes on average tens of thousands to hundreds of thousands of cells, CyTOF achieves a higher throughput (on average up to millions of cells) and classifies cells from a mixture into distinct subtypes based on expression levels of their surface antigen. Cells are first stained by antibodies labeled with metal isotopes and then travel through a time-of-flight mass spectrometer, where the density of each isotope label is quantified [2]. Compared with traditional flow cytometry, which utilizes fluorescent labels, CyTOF overcomes the issues of spectral overlap and autofluorescence, enabling biologists to obtain high-dimensional protein analysis on the single-cell level within the same experimental batch [9].

The rapid advance in experimental technologies inevitably introduces many challenges for data processing and analysis. One key task of mass cytometry data analysis is the investigation of functionally distinct cell populations in high-dimensional spaces [10]. Conventionally, the identification of cell population is achieved by “manual gating,” which is manually defining distinct cell populations on a series of bi-axial plots (dot plots showing the expression of two proteins for all cells) base on prior knowledge [2, 11, 12]. This labor-intensive method provides slow but accurate cell classification. In some cases, this prior knowledge is considered “ground truth” and is used to develop a semi-supervised classifier. For example, Automated Cell-type Discovery and Classification (ACDC) [13] utilizes a marker × cell type annotation table to define landmark points for all populations, then links the remaining cells to these landmarks using random walking. Another linear algorithm called linear discriminant analysis (LDA) [11] also achieves high clustering precision with predetermined manual labels.

An alternative strategy to identify cell populations is to automatically partition cells according to the data structure, regardless of prior knowledge. A handful of mathematical model-based unsupervised clustering tools have been developed for this purpose [12]. Among the different algorithms for processing high-dimensional data, t-distributed Stochastic Neighbor Embedding (t-SNE) is a mainstream method for dimension reduction and data visualization [14] and is widely used in the area of single-cell analysis. Many clustering tools have been developed with t-SNE embedded in their functionalities. Clustering methods, such as Accense [15] and ClusterX [16], carry out density estimation and cluster partitioning on the 2D projection of t-SNE, while others, such as viSNE [17] and PhenoGraph [18], include t-SNE only for visualization. Since CyTOF data do not have as many dimensions as other single-cell data, such as scRNA-seq data, many clustering approaches do not contain a dimension reduction step. The classic clustering method, kmeans, which has been applied to the analysis of CyTOF data [19, 20], can directly group cells into clusters with a minimum within-cluster sum of squares in high-dimensional spaces. Other algorithms that partition cells based on local density also estimate the density distribution in original high-dimensional spaces [12, 13], though they visualize the distribution on a 2D projection of t-SNE. Two popular clustering tools, PhenoGraph [18] and Xshift [21], utilize the *k*-nearest neighbors (KNN) [22] technique to detect connectivity and density peaks among cells embedded in high-dimensional spaces [23, 24].

Since various clustering methods have been used in many different CyTOF data analyses, researchers are often overwhelmed when selecting a suitable clustering method to analyze CyTOF data. There have been a few efforts devoted to comparing some existing tools, but they mainly focus on accuracy [25] or stability [26], providing comparison results based on various aspects of clustering performance. The performance aspects considered in previous literature can offer some guidance in choosing a suitable tool for CyTOF analysis; however, some vital problems remain unevaluated: Do the characteristics of the dataset impact clustering method choice? What is the difference between unsupervised and semi-supervised methods? How does one balance the tradeoffs among cluster performance, stability, and efficiency (runtime)? Answering such questions requires the inclusion of more heterogeneous datasets and more indicators that measure the performance of cluster analysis from multiple aspects.

To address these challenges, we compared the performance of nine popular clustering methods (Table 1) in three categories—precision, coherence, and stability—using six independent datasets (Additional file 1: Figure S1). This comparison would allow cytometry scientists to choose the most appropriate tool with clear answers to the following questions: (1) How does one select between unsupervised and semi-supervised tools? (2) How does one choose the most suitable unsupervised or semi-supervised tool in its category?

**Table 1:** Methods compared in the study

Methods		Implementation tools	Description	Ref
Unsupervised	Accense	MATLAB	tSNE dimension reduction and 2D projection, kernel-based estimation of density, density-based peak-finding and partitioning	[15]
	PhenoGraph	R (cytofkit package)	Detection of *k*-nearest neighbors of each cell, Jaccard similarity coefficient as connectivity, community detection based on connection density	[18]
	Xshift	Vortex	Weighted *k*-nearest neighbor density estimation, detection of density centroids, cells linked to centroid via density-ascending paths	[21]
	FlowSOM	R	Self-organizing map (SOM) trained on scaled data, nodes of SOM connected by minimal spanning tree, consensus hierarchical meta-clustering of nodes	[27]
	flowMeans	R	*K* estimated by peak numbers of kernel density, kmeans clustering of estimated *K*, merging clusters by distance metrics	[20]
	DEPECHE	R	Tuning penalty by resampling dataset, penalized kmeans clustering	[19]
	kmeans	MATLAB	Standard kmeans procedure	
Semi-supervised	ACDC	Python	Marker × cell matrix and cell type × marker table, detect landmark points by community detection, link cells to landmarks by random walkers	[13]
	LDA	MATLAB	Linear discriminant analysis with training datasets	[11]

## Results

To perform a comprehensive investigation on all nine methods, we defined three types of performance assessment categories (Additional file 1: Figure S1): “precision” as external evaluation, “coherence” as internal evaluation, and stability. All clustering methods were investigated on six CyTOF datasets: three well-annotated bone marrow datasets (Levine13dim, Levine32dim, Samusik01) [18, 21], two datasets for muscle cells [28] and in vitro cell lines (Cell Cycle) [29], and one of our own experimental datasets on colon cancer (see the “Methods” section, Additional file 1: TableS1). The performance evaluation procedure was carried out in the following sequential logic, which can be summarized into three parts:

1. For the “precision” as external evaluation assessment, regarding the manually gated labels as “ground truth” as performed by Weber and Robinson [25], we separately explored the performances of semi-supervised and unsupervised tools. Meanwhile, we analyzed the efficiency of each compared tool.
2. For the “coherence” as internal evaluation assessment, we no longer took manually gated labels into account, and directly discussed the ability of each tool to identify the inner structure of data sets by three internal indicators. In this part, since no manually gated labels were considered, we could compare semi-supervised and unsupervised tools between each other.
3. For the stability assessment, we explored the robustness of each tool on clustering accuracy and the identified number of clusters, in terms of varying sampling sizes. Based on the results of stability evaluation for the number of identified clusters, we further evaluated the extended question of clustering resolution. Finally, we integrated the analysis results to provide a clear guidance for tool selection.

Before our analysis began, we encountered the problem that different tools recommend distinct data transformation procedures and the impact of different procedures on clustering results has not been thoroughly analyzed. Thus, we applied five popular transformation procedures (Additional file 1: Supplementary methods) on the colon dataset, consolidated them into one optimal procedure, and used this procedure throughout our study. As shown in Additional file 1: Table S2, both the classic arcsinh procedure and its two modified versions (raw data minus one before arcsinh transformation then set negative values to zero, or a randomized normal distribution) yielded similar clustering results across various tools. Compared with the two modified procedures, the classic arcsinh transformation provided a higher precision for flowMeans. The Logicle transformation and 0–1 scaling, two procedures widely applied in the field of flow cytometry [20], led to relatively poor results for mass cytometry data in our analysis. Taken together, we decided to process all the datasets using an arcsinh transformation with a co-factor of 5 (see the “Methods” section), and we did not use any of the other transformation options that had previously been implemented in all of the tools we tested.

### External evaluations of semi-supervised tools suggest that LDA is the preferred semi-supervised tool in terms of precision

We started the analysis by evaluating the ability to reproduce manual labels. This was achieved by evaluating our first performance assessment category, the “precision,” as external evaluation, using four indicators (see the “Methods” section) on all nine clustering methods (Table 1): accuracy, weighted *F*-measure, Normalized Mutual Information (NMI), and Adjusted Rand Index (ARI) [30, 31].

**Fig. 1:**
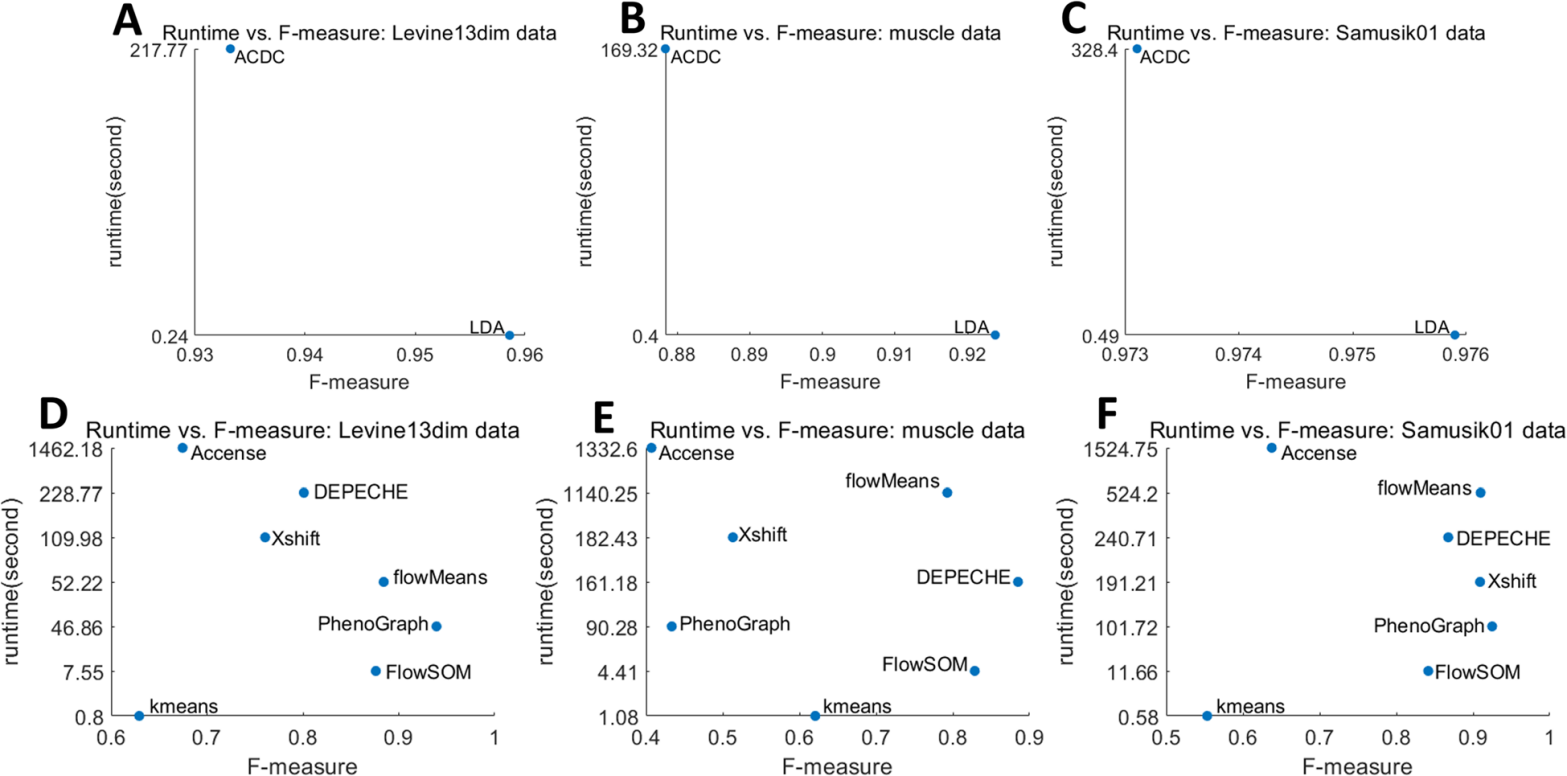
Runtime and *F*-measure of semi-supervised tools (**a**–**c**) and unsupervised tools (**d**–**f**) on Levine13dim, muscle, and Samusik01 datasets

Table 2 summarizes the comparison results of semi-supervised methods. As expected, the two semi-supervised methods showed better performance than unsupervised methods (Table 3). In all datasets, both ACDC and LDA had greater accuracy, *F*-measure, NMI, and ARI than all unsupervised methods. This observation is most noticeable in Cell Cycle data (*F*-measure > 0.82 vs. *F*-measure = 0.2–0.68), where the number of features [32] is significantly larger than the number of labels [4]. Next, we found that in all datasets except for Levine32dim, LDA had moderately better performance than ACDC. The significant lower runtime of LDA (Fig. 1 and Additional file 1: Figure S2) also indicates that LDA may be the top choice for the task of reproducing manual labels.

**Table 2:** Summary of external evaluations for semi-supervised methods

Datasets	Methods	External evaluations			
		Accuracy	*F*-measure	NMI	ARI
Cell Cycle	ACDC	0.8342 ± 0.0071	0.8466 ± 0.0093	0.4325 ± 0.0212	0.5579 ± 0.0129
	LDA	0.9095 ± 0.0006	0.9110 ± 0.0005	0.6189 ± 0.0032	0.7225 ± 0.0021
Colon	ACDC	0.7439 ± 0.0026	0.7874 ± 0.0076	0.5705 ± 0.0088	0.5952 ± 0.0041
	LDA	0.8576 ± 0.0011	0.8587 ± 0.0012	0.7410 ± 0.0012	0.7626 ± 0.0017
Levine13dim	ACDC	0.9010 ± 0.0029	0.9275 ± 0.0026	0.8635 ± 0.0041	0.9011 ± 0.0052
	LDA	0.9582 ± 0.0005	0.9586 ± 0.0005	0.9275 ± 0.0008	0.9539 ± 0.0007
Levine32dim	ACDC	0.9943 ± 0.0006	0.9939 ± 0.0007	0.9380 ± 0.0052	0.9791 ± 0.0020
	LDA	0.9809 ± 0.0003	0.9807 ± 0.0004	0.9595 ± 0.0006	0.9830 ± 0.0002
Muscle	ACDC	0.8787 ± 0.0101	0.8784 ± 0.0089	0.6750 ± 0.0168	0.7593 ± 0.0190
	LDA	0.9240 ± 0.0011	0.9238 ± 0.0011	0.7606 ± 0.0031	0.8295 ± 0.0031
Samusik01	ACDC	0.9682 ± 0.0027	0.9731 ± 0.0019	0.9347 ± 0.0047	0.9616 ± 0.0021
	LDA	0.9757 ± 0.0002	0.9759 ± 0.0002	0.9482 ± 0.0004	0.9735 ± 0.0005

Data shown as mean ± standard deviation

**Table 3:** Summary of external evaluations for unsupervised methods

Datasets	Methods	External evaluations			
		Accuracy	*F*-measure	NMI	ARI
Cell Cycle	Accense	0.3529 ± 0.0471	0.3500 ± 0.0636	0.2490 ± 0.0240	0.1682 ± 0.0395
	PhenoGraph	0.2309 ± 0.0268	0.2789 ± 0.0196	0.1364 ± 0.0087	0.0683 ± 0.0074
	Xshift	0.3622 ± 0.0419	0.3970 ± 0.0383	0.1710 ± 0.0165	0.0752 ± 0.0353
	kmeans	0.3969 ± 0.0021	0.4224 ± 0.0019	0.0963 ± 0.0016	0.0681 ± 0.0016
	flowMeans	0.3055 ± 0.0061	0.3506 ± 0.0051	0.1849 ± 0.0047	0.0694 ± 0.0058
	FlowSOM	0.6605 ± 0.0021	0.6897 ± 0.0029	0.1040 ± 0.0035	0.1171 ± 0.0025
	DEPECHE	0.2808 ± 0.0126	0.3551 ± 0.0110	0.1361 ± 0.0246	0.0546 ± 0.0197
Colon	Accense	0.3209 ± 0.0353	0.3495 ± 0.0442	0.4504 ± 0.0286	0.2304 ± 0.0304
	PhenoGraph	0.3772 ± 0.0201	0.3994 ± 0.0137	0.4254 ± 0.0073	0.2696 ± 0.0094
	Xshift	0.3187 ± 0.0167	0.3094 ± 0.0124	0.3763 ± 0.0076	0.2399 ± 0.0299
	kmeans	0.4480 ± 0.0216	0.4951 ± 0.0086	0.4688 ± 0.0073	0.3235 ± 0.0132
	flowMeans	0.5095 ± 0.0894	0.5901 ± 0.0582	0.4705 ± 0.0619	0.3724 ± 0.1125
	FlowSOM	0.5597 ± 0.0284	0.5888 ± 0.0230	0.5303 ± 0.0157	0.4157 ± 0.0288
	DEPECHE	0.5902 ± 0.0186	0.6898 ± 0.0132	0.4694 ± 0.0239	0.5042 ± 0.0188
Levine13dim	Accense	0.6055 ± 0.0946	0.6745 ± 0.0929	0.7486 ± 0.0603	0.6408 ± 0.1034
	PhenoGraph	0.8880 ± 0.0015	0.9123 ± 0.0167	0.8639 ± 0.0078	0.8884 ± 0.0159
	Xshift	0.7573 ± 0.0091	0.7606 ± 0.0125	0.7359 ± 0.0118	0.7465 ± 0.0116
	kmeans	0.5684 ± 0.0504	0.6293 ± 0.0325	0.7070 ± 0.0193	0.5721 ± 0.0465
	flowMeans	0.8470 ± 0.0486	0.8842 ± 0.0343	0.8352 ± 0.0282	0.8349 ± 0.0459
	FlowSOM	0.8540 ± 0.0235	0.8760 ± 0.0200	0.8590 ± 0.0097	0.8732 ± 0.0168
	DEPECHE	0.6929 ± 0.0142	0.8010 ± 0.0077	0.6687 ± 0.0099	0.6571 ± 0.0118
Levine32dim	Accense	0.5514 ± 0.0794	0.6008 ± 0.0627	0.6876 ± 0.0389	0.5289 ± 0.0740
	PhenoGraph	0.6369 ± 0.0253	0.7062 ± 0.0213	0.7410 ± 0.0142	0.6437 ± 0.0240
	Xshift	0.7543 ± 0.0605	0.7706 ± 0.0469	0.7690 ± 0.0323	0.7593 ± 0.0737
	kmeans	0.5753 ± 0.0413	0.6748 ± 0.0231	0.7302 ± 0.0115	0.6405 ± 0.0638
	flowMeans	0.9216 ± 0.0318	0.9279 ± 0.0337	0.9115 ± 0.0290	0.9397 ± 0.0342
	FlowSOM	0.8787 ± 0.0975	0.8979 ± 0.0664	0.8840 ± 0.0600	0.8803 ± 0.1267
	DEPECHE	0.8931 ± 0.0010	0.9231 ± 0.0009	0.8436 ± 0.0008	0.9297 ± 0.0009
Muscle	Accense	0.3687 ± 0.0663	0.4072 ± 0.0743	0.3953 ± 0.0391	0.2397 ± 0.0759
	PhenoGraph	0.3930 ± 0.0325	0.4336 ± 0.0249	0.3912 ± 0.0083	0.2694 ± 0.0261
	Xshift	0.5119 ± 0.0591	0.5133 ± 0.0463	0.3882 ± 0.0190	0.3572 ± 0.0405
	kmeans	0.6113 ± 0.0046	0.6207 ± 0.0045	0.4958 ± 0.0030	0.4243 ± 0.0055
	flowMeans	0.7880 ± 0.0193	0.7928 ± 0.0089	0.5841 ± 0.0145	0.6364 ± 0.0347
	FlowSOM	0.8210 ± 0.0068	0.8286 ± 0.0073	0.6470 ± 0.0072	0.6688 ± 0.0175
	DEPECHE	0.8346 ± 0.0018	0.8850 ± 0.0019	0.5792 ± 0.0015	0.7074 ± 0.0035
Samusik01	Accense	0.5868 ± 0.0502	0.6376 ± 0.0400	0.7165 ± 0.0237	0.5574 ± 0.0290
	PhenoGraph	0.9260 ± 0.0412	0.9249 ± 0.0344	0.8979 ± 0.0285	0.9250 ± 0.0526
	Xshift	0.8909 ± 0.0485	0.9091 ± 0.0324	0.8742 ± 0.0196	0.8781 ± 0.0561
	kmeans	0.4837 ± 0.0572	0.5535 ± 0.0401	0.6437 ± 0.0186	0.4655 ± 0.0482
	flowMeans	0.9064 ± 0.0163	0.9099 ± 0.0163	0.8818 ± 0.0137	0.9206 ± 0.0045
	FlowSOM	0.8386 ± 0.0693	0.8417 ± 0.0668	0.8185 ± 0.0639	0.8561 ± 0.0732
	DEPECHE	0.8300 ± 0.0047	0.8677 ± 0.0047	0.7271 ± 0.0050	0.8298 ± 0.0068

Data shown as mean ± standard deviation

Although LDA is superior to ACDC in terms of precision, we all know that the precision of semi-supervised tool relies more on the availability of prior information. Since a training set is only necessary for LDA but not for ACDC, which requires a “marker × cell type” table instead, it is questionable whether LDA can still outperform ACDC when the training set is less sufficient. To answer this question, we first trained LDA with only a limited proportion of samples (randomly choosing 20%, 40%, 60%, and 80% of all samples in colon dataset) as the training set. We observed that the performance of LDA stayed constant when the size of training set varied (Additional file 1: Figure S3). Then, we trained LDA with all the cells from healthy colon tissue in the colon dataset, and predicted the labels of all the remaining cells from polyps, early-stage cancer tissue, and late-stage cancer tissue. We then applied ACDC to the entire colon dataset as well as the subset excluding cells from healthy tissue (Additional file 1: Figure S3). The predicted result from LDA was then compared to that from ACDC. Under these conditions, the *F*-measure of LDA dropped from 0.85 to 0.73, which was not better than that of ACDC (0.80 for the entire dataset, 0.74 for the subset excluding cells from healthy tissue). Similar tests were repeated on the Cell Cycle dataset with consistent results (Additional file 1: Figure S3): when only one cell line (THP, HELA, or 293 T) was chosen as the training set, LDA could not precisely classify samples from other cell lines. Thus, we concluded that LDA can be regarded as the optimal semi-supervised tool as long as the training set and the test set are homogenous.

### External evaluations of unsupervised tools highlight the precision of FlowSOM and flowMeans

Next, we performed external evaluation for seven unsupervised methods and observed that the precisions of different tools varied among different datasets. Compared to other methods, FlowSOM had relatively high precision values among all datasets (Table 3). In the Cell Cycle dataset, FlowSOM was the only unsupervised tool that had an *F*-measure larger than 0.5. FlowSOM also had a relative short runtime (Fig. 1 and Additional file 1: Figure S2), which is another advantage to be considered when choosing a suitable tool. In other datasets, such as the muscle and colon datasets (Table 3), flowMeans had similar precision to FlowSOM. In fact, flowMeans outperformed FlowSOM in Samusik01 data (ARI 0.92 vs. 0.85). However, PhenoGraph had the best performance in the Levine13dim (ARI 0.927) and Samusik01 (ARI 0.925) datasets but performed poorly in the muscle, Cell Cycle, and colon datasets. On the contrary, DEPECHE exhibited excellent performance in datasets with relatively small numbers of cell types such as Levine32dim (*F*-measure = 0.92), muscle (*F*-measure = 0.89), and colon (*F*-measure = 0.68). In summary, FlowSOM and flowMeans had overall better precisions in our external evaluation, followed by PhenoGraph and DEPECHE.

### Internal evaluations indicate that DEPECHE, FlowSOM, and PhenoGraph best captured the inner structure of CyTOF data

We have exploited external evaluation metrics to analyze whether a clustering tool can accurately reproduce the manual-gated labels as the “ground truth.” However, researchers often wish to partition cells based on the natural structure of biomarker expression profile without considering any assumptions about cell partitions. Here, we analyzed the ability of a clustering tool to detect the inner structure of each dataset for the “coherence” assessment using three internal evaluations [33]—the Calinski-Harabasz index (CH, larger is better), Davies-Bouldin index (DB, smaller is better), and Xie-Beni index (XB, smaller is better)—in contrast to checking for reproducibility of sets of manual-gated labels by each tool. The detailed description of these indices is presented in the “Methods” section. These three internal evaluations have all been defined based on the assumption that an ideal cell partition should have both high within-group similarity and high between-group dissimilarity, which is exactly the characteristic that the natural clustering structure of CyTOF data should exhibit.

Table 4 shows that DEPECHE had noticeably high CH and low DB indices in all datasets and outperformed nearly all other tools. However, this observation should be interpreted with caution: CH and DB are indices that naturally favor kmeans-based algorithms [33], and the simple kmeans clustering also achieved high performance based on CH and DB. Aside from DEPECHE and kmeans, PhenoGraph and FlowSOM also demonstrated good internal evaluation results over different datasets. PhenoGraph had the highest CH (larger is better), lowest DB (smaller is better), and third-lowest XB (smaller is better) in both the Levine13dim and Samusik01 datasets, whereas FlowSOM had the highest CH, lowest DB, and second-lowest XB in both the muscle and Cell Cycle datasets. In contrast to the above tools with consistent good results on all three indices, we observed inconsistency in the performance of Accense: it had the lowest XB in the Levine13dim, muscle, Cell Cycle, and colon datasets but showed poor performance with respect to CH and DB. We reasoned that this inconsistency might be because XB naturally favors density-based algorithms [33]; hence, there is currently not enough evidence to state that Accense gives coherent clustering results.

**Table 4:** Summary of internal evaluations for each compared methods

Datasets	Methods	Internal evaluations		
		CH	DB	XB
Cell Cycle	Accense	3.2409 ± 0.2069	2.4746 ± 0.2563	0.6473 ± 0.0750
	PhenoGraph	3.4250 ± 0.0350	2.5692 ± 0.1503	0.7864 ± 0.0392
	Xshift	3.5620 ± 0.0443	1.7337 ± 0.1618	0.9131 ± 0.3066
	kmeans	3.9414 ± 0.0011	1.5971 ± 0.0013	0.7705 ± 0.0165
	flowMeans	3.5992 ± 0.0283	1.6237 ± 0.0341	0.7294 ± 0.0393
	FlowSOM	3.6294 ± 0.0036	1.3578 ± 0.0351	0.6669 ± 0.0269
	DEPECHE	3.8372 ± 0.0283	1.7767 ± 0.0322	0.7695 ± 0.0177
	ACDC	3.4645 ± 0.0901	2.0773 ± 0.3156	0.9395 ± 0.0428
	LDA	3.4581 ± 0.0036	2.5080 ± 0.0191	1.0021 ± 0.0358
Colon	Accense	3.2688 ± 0.0622	1.7064 ± 0.0656	1.0427 ± 0.0545
	PhenoGraph	3.4231 ± 0.0194	1.9030 ± 0.0245	1.3997 ± 0.1199
	Xshift	3.0706 ± 0.0179	2.3218 ± 0.0595	1.3207 ± 0.0511
	kmeans	3.5109 ± 0.0079	1.9037 ± 0.0501	1.4570 ± 0.1055
	flowMeans	3.4793 ± 0.1068	1.5864 ± 0.0421	1.6706 ± 0.1958
	FlowSOM	3.6435 ± 0.0117	1.6583 ± 0.0514	1.5910 ± 0.1568
	DEPECHE	3.9819 ± 0.0375	1.7725 ± 0.0250	1.6325 ± 0.0930
	ACDC	3.5898 ± 0.0440	2.0607 ± 0.1160	1.4239 ± 0.0754
	LDA	3.5100 ± 0.0009	2.1773 ± 0.0065	1.5564 ± 0.0724
Levine13dim	Accense	3.4230 ± 0.0932	1.8832 ± 0.1408	1.2321 ± 0.0278
	PhenoGraph	4.0739 ± 0.0176	1.4645 ± 0.0346	1.3972 ± 0.1349
	Xshift	3.5106 ± 0.0289	2.4284 ± 0.0443	1.7868 ± 0.0476
	kmeans	3.8508 ± 0.0150	2.1550 ± 0.0546	1.6213 ± 0.1471
	flowMeans	4.0475 ± 0.0194	1.5030 ± 0.0849	1.4234 ± 0.1182
	FlowSOM	3.8486 ± 0.0071	1.7564 ± 0.0615	1.5043 ± 0.1531
	DEPECHE	4.2783 ± 0.0174	1.1677 ± 0.0342	1.3562 ± 0.0392
	ACDC	3.9638 ± 0.0110	1.4916 ± 0.0370	1.3109 ± 0.0948
	LDA	3.8288 ± 0.0106	2.0046 ± 0.0493	1.3828 ± 0.1167
Levine32dim	Accense	3.4621 ± 0.0901	2.3414 ± 0.0925	0.7891 ± 0.0950
	PhenoGraph	3.7401 ± 0.0081	1.8293 ± 0.0810	1.0009 ± 0.0479
	Xshift	3.6669 ± 0.0102	2.2576 ± 0.1324	0.8295 ± 0.1382
	kmeans	3.8761 ± 0.0166	2.0587 ± 0.0386	0.9972 ± 0.0441
	flowMeans	3.8546 ± 0.0393	1.6975 ± 0.2199	0.7985 ± 0.0709
	FlowSOM	3.8244 ± 0.0285	1.5974 ± 0.0863	0.8366 ± 0.0792
	DEPECHE	4.1480 ± 0.0009	1.4727 ± 0.0023	0.7575 ± 0.0351
	ACDC	3.6169 ± 0.0046	1.3974 ± 0.0049	0.7693 ± 0.1310
	LDA	3.8297 ± 0.0007	1.7011 ± 0.0099	0.7155 ± 0.0139
Muscle	Accense	3.2254 ± 0.1688	2.3190 ± 0.3178	0.8420 ± 0.1211
	PhenoGraph	3.6052 ± 0.0132	1.8619 ± 0.0417	1.7228 ± 0.2389
	Xshift	3.2898 ± 0.0274	2.2460 ± 0.1524	2.1455 ± 0.1599
	kmeans	3.9722 ± 0.0022	1.7729 ± 0.0099	1.4987 ± 0.1212
	flowMeans	3.3809 ± 0.0565	1.7685 ± 0.0769	1.3750 ± 0.1428
	FlowSOM	3.8262 ± 0.0146	1.4439 ± 0.0462	1.3586 ± 0.1473
	DEPECHE	4.2639 ± 0.0044	1.2235 ± 0.0080	1.2892 ± 0.0269
	ACDC	3.6900 ± 0.0305	1.7186 ± 0.0515	1.5641 ± 0.15555
	LDA	3.8073 ± 0.0031	1.6641 ± 0.0206	1.6003 ± 0.0900
Samusik01	Accense	3.2952 ± 0.0476	2.3424 ± 0.1454	0.6500 ± 0.0266
	PhenoGraph	3.7380 ± 0.0033	1.4607 ± 0.0424	0.7669 ± 0.0831
	Xshift	3.2660 ± 0.0134	2.7855 ± 0.4644	1.2670 ± 0.1654
	kmeans	3.7087 ± 0.0135	2.2072 ± 0.0854	0.9365 ± 0.0792
	flowMeans	3.4029 ± 0.0381	1.6069 ± 0.2374	0.6309 ± 0.0475
	FlowSOM	3.6941 ± 0.0257	1.8967 ± 0.3799	0.8286 ± 0.0832
	DEPECHE	4.1028 ± 0.0021	1.4867 ± 0.0054	0.9141 ± 0.0109
	ACDC	3.6827 ± 0.0178	1.3871 ± 0.0132	0.7273 ± 0.0573
	LDA	3.6767 ± 0.0080	1.6325 ± 0.0242	1.0414 ± 0.0808

Data shown as mean ± standard deviation. *CH* Calinski-Harabasz index (log10 transformed), *DB* Davies-Bouldin index, *XB* Xie-Beni index (log10 transformed)

A noteworthy fact is that unlike their strength in external evaluation, semi-supervised tools no longer ranked top with respect to any of the internal evaluation indices. This result is consistent with the fact that even the manual labels themselves did not perform as well as top unsupervised tools in internal evaluation (Additional file 1: Table S3). Compared to LDA, ACDC showed better performance in internal evaluation. In some cases (DB and XB for Samusik01 and Levine32dim, DB for Levine13dim, etc.), the performance of ACDC was comparable with that of top-ranking unsupervised tools.

Given the above analysis, we recommended FlowSOM, PhenoGraph, and DEPECHE as preferred tools for the task of capturing inner structure of CyTOF data.

### Stability evaluations suggest that PhenoGraph, DEPECHE, and LDA exhibited high robustness

We have described the performances of nine tools from two perspectives: external evaluations (i.e., precision) and internal evaluations (i.e., coherence). Next, we investigated the stability performance of different tools. We firstly tested the robustness on the clustering precision and coherence of nine tools under two separate conditions: (1) given a fixed sample size, but with different subsampling datasets, for testing; (2) directly given different subsampling sizes, ranging from 5000 cells to 80,000 cells, for testing. Then, we explored the robustness of each tool with respect to the number of identified clusters with varying sampling sizes.

**Fig. 2:**
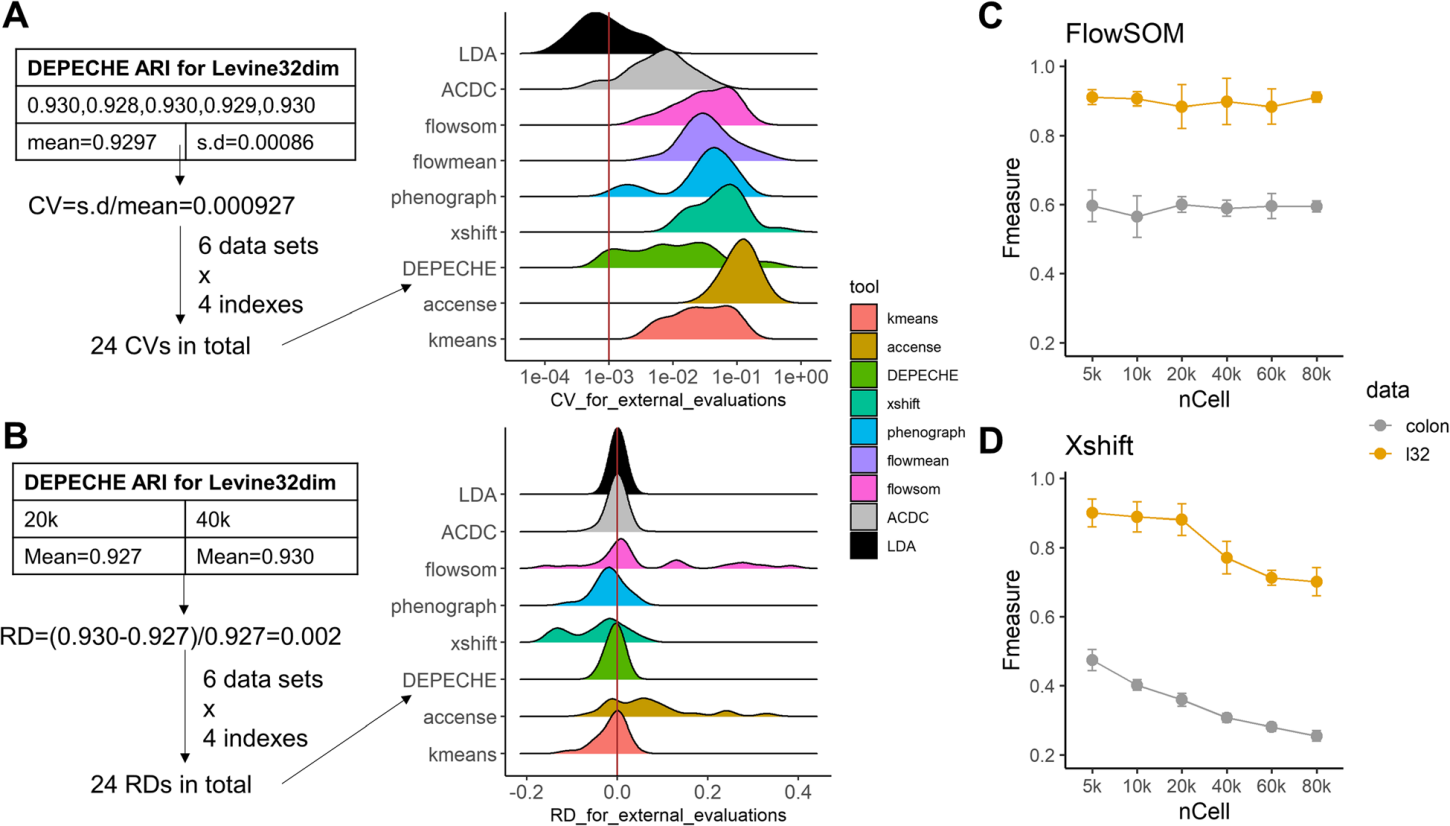
Stability of each tool. **a** Left: schematic diagram showing how coefficients of variation (CVs) were calculated and integrated; right: distribution of CVs for external evaluations for each tool. The red solid line represents median CV for LDA, which is the smallest median CV. **b** Same as **a**, but shows distribution of relative difference (RD) between 20,000 cell and 40,000 cell subsampling tests. The red solid line marks RD = 0, which means that there is no difference in performance between 20,000 cell and 40,000 cell subsampling tests. **c** Precision of FlowSOM on Levine32dim and colon datasets changed as sample size changed. **d** Same as **c**, but for Xshift

When considering the performance of a clustering tool, although its ability to cluster data into different meaningful populations is of great significance, its stability (or robustness) is also important. Therefore, we measured the robustness against a fixed subsampling size by using the coefficient of variation (CV, smaller indicates better stability), and we measured the robustness against varying sample sizes by using the relative difference (RD, close to zero indicates better stability) between 20,000 cell tests (Additional file 2) and 40,000 cell tests (Tables 2, 3, and 4, also see the “Methods” section). As shown in Fig. 2a and Additional file 1: Figure S4A, both semi-supervised tools and top-performing unsupervised tools had a high robustness against random subsampling: median CVs for external evaluation in all datasets ranged from 0.001 (LDA) to 0.054 (Xshift), whereas those for internal evaluation ranged from 0.010 (LDA and DEPECHE) to 0.049 (flowMeans). A few extreme CV values for Xshift (ARI in CC data 0.46), DEPECHE (ARI in CC data 0.36), and flowMeans (ARI in colon data 0.31) indicate that performance of these tools might decline in specific cases. Thus, we observed that LDA had the best stability (largest CV for external evaluation < 0.006; largest CV for internal evaluation = 0.08), followed by PhenoGraph (largest CV for external evaluation = 0.11; largest CV for internal evaluation < 0.14).

By comparing the impact of varying sampling sizes on each tool (Fig. 2b and Additional file 1: Figure S4B), we observed that LDA, ACDC, DEPECHE, and PhenoGraph did not have large differences when the sample size expanded from 20,000 to 40,000. They all had a relative difference (RD, see the “Methods” section) close to zero for all datasets. Xshift and FlowSOM exhibited some instability: the distribution of RD for Xshift was biased toward negative numbers, indicating that the precision of Xshift declined as sample size grew large. Although RD of FlowSOM was consistently around zero, there were some extreme values: RD for ARI in Samusik01 data was 0.38, whereas that in muscle data was 0.27. Similar results were obtained from RD of internal evaluation metrics (Additional file 1: Figure S4B). Since flowMeans frequently introduced singularity errors with a sample size of less than or equal to 20,000 (data not shown), we did not consider testing on flowMeans.

To further investigate the influence of sample size on Xshift and FlowSOM, we carried out additional subsampling tests (random sampling of 5000, 10,000, 60,000, and 80,000 cells). In both the Levine32dim and colon datasets, *F*-measure of Xshift dropped significantly as the sample size grew large. Although average *F*-measure of FlowSOM was relatively stable across different sample sizes, the standard deviation of *F*-measure reached a minimum when sample size reached a maximum (80,000 cells in both datasets), indicating that FlowSOM was more robust at analyzing large datasets (Fig. 2c, d).

### PhenoGraph and Xshift detect more clusters, especially with a large sample size

We believed that the robustness of a method should be evaluated by the stability of not only the performance of clustering but also the number of identified clusters. Therefore, we further explored the robustness of methods with respect to the number of identified clusters with varying sampling sizes. Since four of the tested tools (ACDC, LDA, kmeans, and FlowSOM) take the number of clusters as a required known input, we only investigated the robustness of the other five tools (Accense, PhenoGraph, flowMeans, Xshift, and DEPECHE).

**Fig. 3:**
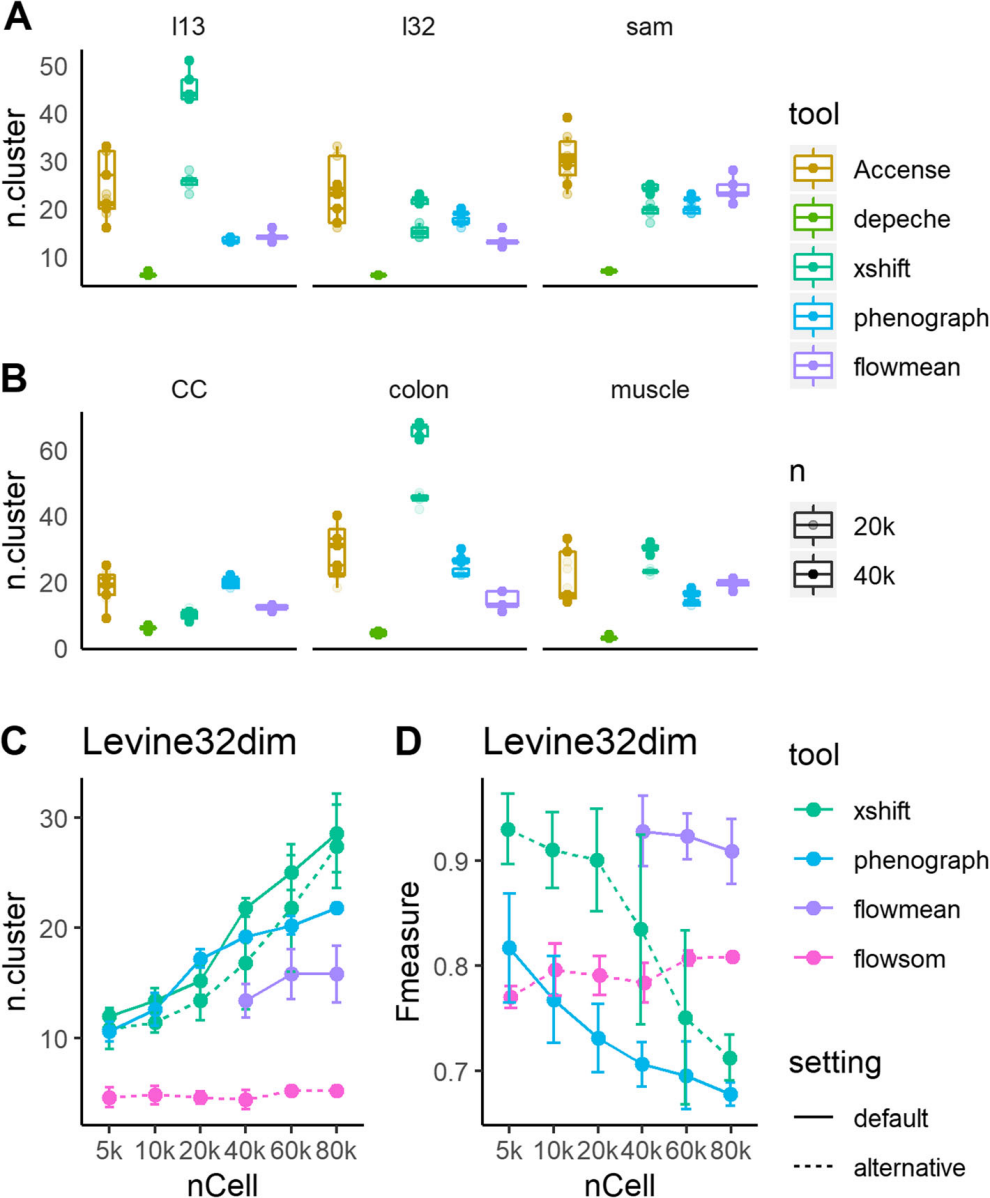
Number of clusters detected by each tool. **a**, **b** Number of clusters detected by default settings of Accense, DEPECHE, Xshift, PhenoGraph, and flowMeans. Translucent points represent results in 20,000 cell subsampling tests. **c**, **d** Number of clusters (**c**) and precision (**d**) of highlighted tools and settings were impacted by sample size in the Levine32dim dataset. Dotted lines represent performance of alternative settings of different tools (Xshift: Elbow Plot Determination; FlowSOM: automatic estimation of number of clusters). Since the precision of the default settings of Xshift and FlowSOM have been shown in Fig. 2, they are not presented here

As shown in Fig. 3a, b, DEPECHE detected a small number of clusters in all datasets with little deviation. In all datasets and sample sizes, the number of clusters identified by DEPECHE ranged between 3 and 8. On the contrary, Xshift detected more clusters compared to all other tools. As the sample size grew from 20,000 to 40,000, the number of clusters identified by Xshift also grew significantly. PhenoGraph also identified a relatively large number of clusters in the Levine32dim, Cell Cycle, and colon datasets and was moderately impacted by sample size in the Samusik01 and colon datasets. The number of clusters detected by flowMeans was not as extreme as DEPECHE or Xshift but was more inconsistent compared to DEPECHE, Xshift, and PhenoGraph in 40,000 cells subsampling tests.

Given that PhenoGraph and Xshift identified more clusters and that flowMeans was more inconsistent than the above two tools, we carried out further subsampling tests for PhenoGraph, Xshift, and flowMeans to evaluate the influence of sample size on robustness. Since Xshift provides an alternative way to determine the parameter *K* in KNN called Elbow Plot Determination, we carried out further Xshift analysis using the Elbow Plot method to see whether it could give a stable result. Similarly, FlowSOM had an alternative option to estimate the number of clusters within a given range; hence, these two cases were also included in the comparison with varying sampling sizes. As shown in Fig. 3 and Additional file 1: Figure S5, the number of clusters detected by Xshift (default fixed *K*) grew greatly as the sample size grew from 5000 to 80,000, and Xshift (with the alternative Elbow Plot setting) partly decreased this growth. However, the number of clusters detected still grew faster when using Xshift with either setting than when using PhenoGraph. Furthermore, for PhenoGraph and Xshift, the increase in the number of clusters accompanied a decline in precision (Fig. 3d). On the contrary, as the sample size grew, the precision for flowMeans declined without a significant change in the number of detected clusters. An interesting phenomenon is that when FlowSOM was forced to automatically determine the number of clusters, it stably identified very few clusters just like DEPECHE did, but its precision was moderately lower than default setting (Fig. 3d vs. Fig. 2c). Comparing Fig. 2c to Fig. 3d, the precision and the stability of FlowSOM consistently reached their peaks when the sampling size was at its maximum (80,000).

### Xshift and PhenoGraph identified refined sub-clusters of major cell types

**Fig. 4:**
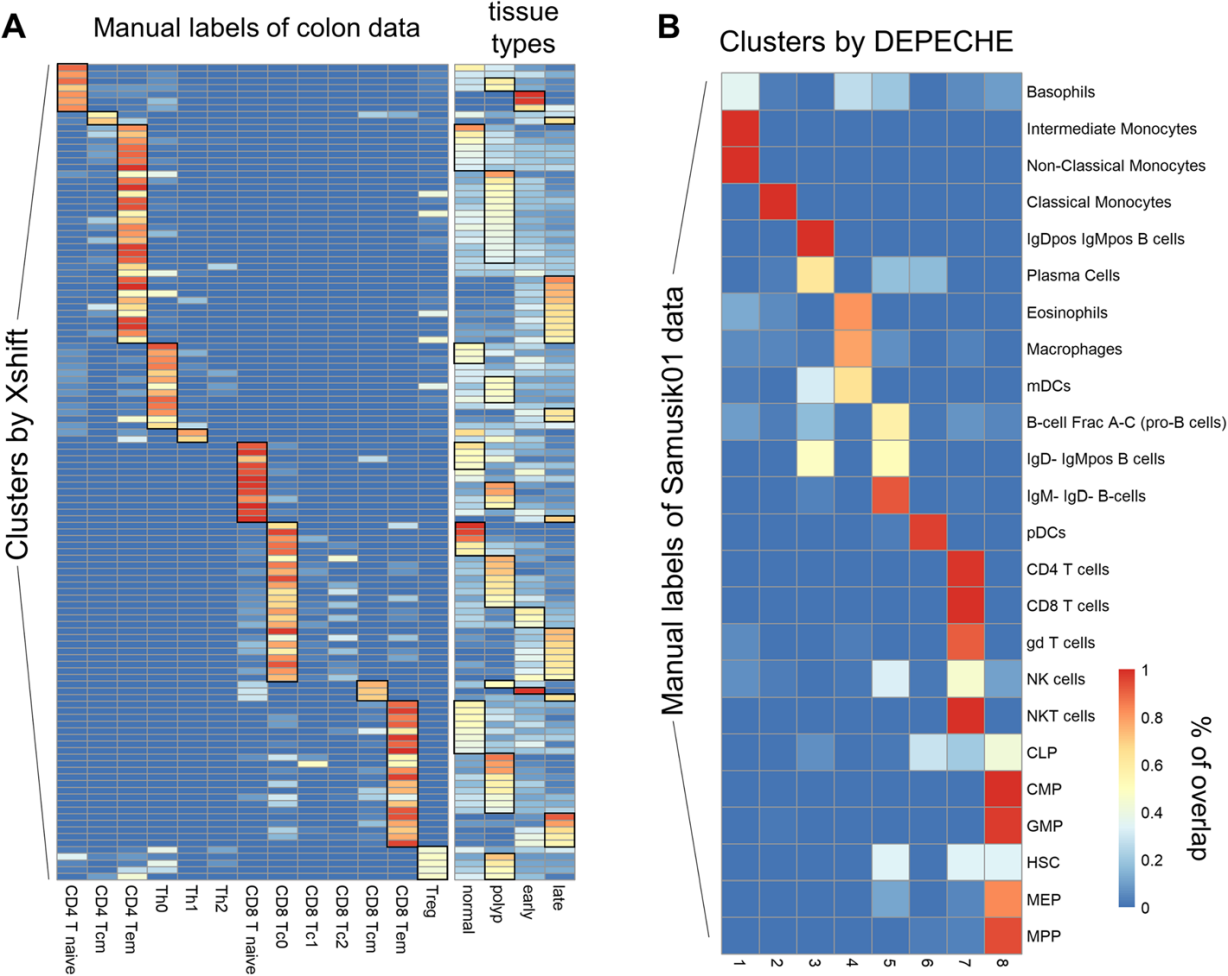
Clustering resolution of Xshift and DEPECHE. **a** Each row represents one cluster detected by Xshift; each column represents a manual label (left panel) of tissue origin (right panel). Color of each grid denotes the proportion of cells of that cluster belonging to the corresponding manual label (left) of tissue (right). Thus, row sums of both panels are 100%. Black grids highlight the specific patterns resolved by Xshift. **b** Similar to **a**, but for the relationship between DEPECHE clusters (column) and manual labels of Samusik01 data (row)

Based on the above comparison analysis, we discovered several notable characteristics of Xshift and PhenoGraph: (1) they had recognizable clustering structures (shown by better internal evaluation results), (2) they tended to overestimate the total number of clusters compared to the number defined by manual gating strategy, and (3) they exhibited reduced precision on datasets that had much smaller numbers of labels than numbers of features (muscle, Cell Cycle, colon). These characteristics suggested that Xshift and PhenoGraph tend to identify refined sub-clusters of major cell types. In other words, if manual gating did not classify these sub-clusters, the predicted clusters from Xshift and PhenoGraph would be very different than the manual labels. To test this hypothesis, we applied Xshift and PhenoGraph on the entire colon dataset and defined a many-to-one alignment between predicted clusters and manual labels: if more than 50% of cells from a predicted cluster belonged to one manual label, we defined that this cluster is a sub-cluster of the corresponding manual label. We found that each of the 132 clusters discovered by Xshift could be aligned to a cell type defined by manual gating (Fig. 4a). This alignment led to an *F*-measure of 0.85, which was much higher than a one-to-one alignment (Table 3). Since colon data involve samples originated from healthy tissue, polyps, early-stage cancer, and late-stage cancer, we tested whether Xshift discovered origin-specific patterns of cell clusters. We found that about three quarters (98 out of 132) of the clusters discovered by Xshift were origin-specific (more than 50% of cells come from the same sample origin) (Fig. 4a). These results demonstrate that Xshift was able to classify specific subtypes of cells. Similar results were also found for PhenoGraph (Additional file 1: Figure S6A). However, since PhenoGraph identified much smaller numbers of clusters than Xshift (34 vs. 132, respectively), its capacity to recognize origin-specific clusters is relatively weaker than that of Xshift.

Next, DEPECHE also has an observable phenomenon that differentiates it from other tools. DEPECHE tended to underestimate the number of clusters and had better precision when the number of manual labels was small. We hypothesize that unlike Xshift and PhenoGraph, DEPECHE tends to group cells into major cell types. Carrying out the same analytical procedure as in Xshift but reversed, we obtained a one-to-many alignment between DEPECHE clusters and the manual labels of the Samusik01 dataset (Fig. 4b). DEPECHE grouped different T cells into one cluster and six types of progenitor cells into another. The difference among subtypes of B cells was also neglected by DEPECHE. We further found that in both the Samusik01 and Levine13dim (Additional file 1: Figure S6B) datasets, DEPECHE failed to recognize the characteristics of some small cell types such as basophil cells, eosinophil cells, nature killer cells, and subtypes of dendritic cells (Additional file 1: Figure S6B). All the above results demonstrate that DEPECHE is not suitable for analyzing refined subtypes.

## Discussion

**Fig. 5:**
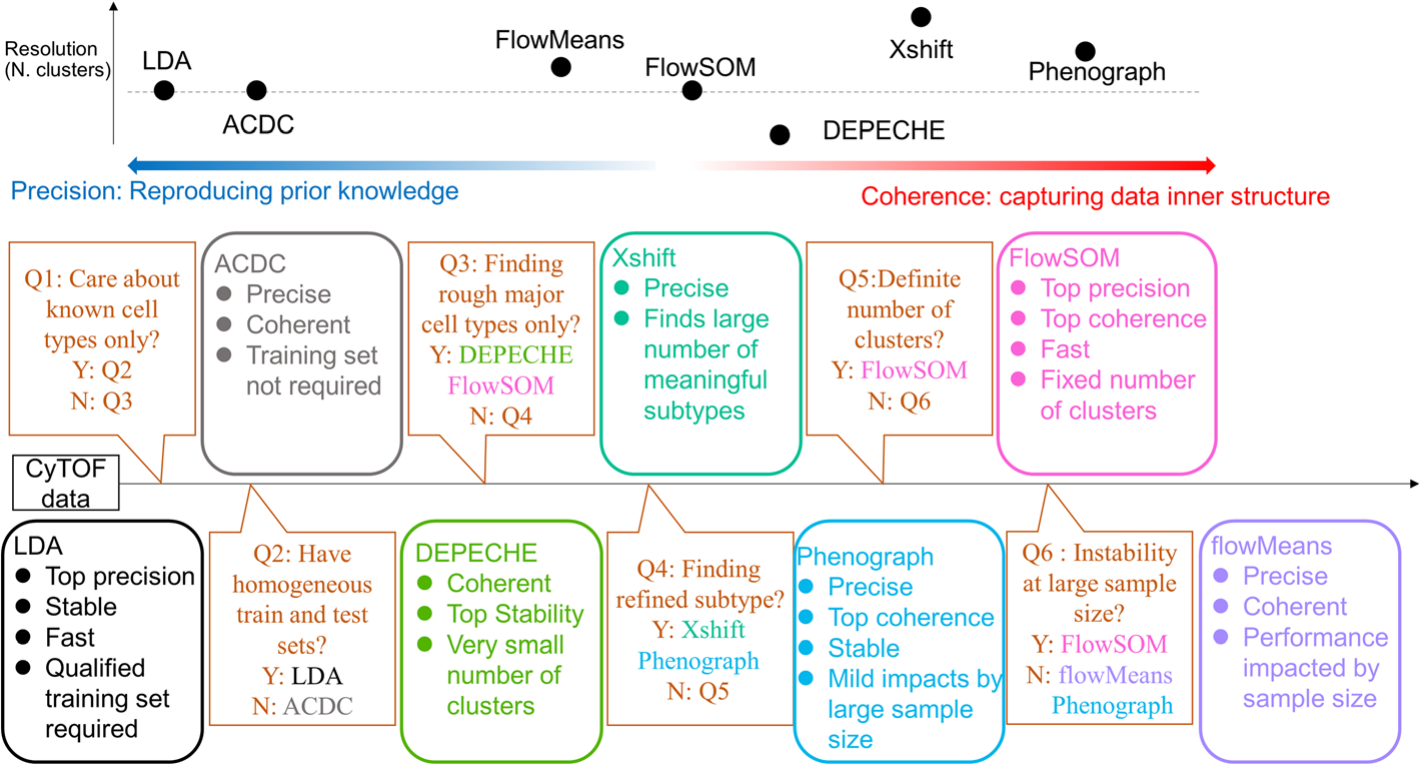
Upper panel: relative advantages of each tool. Note, precision and coherence are not exclusive; some tools like FlowSOM and PhenoGraph are both precise and coherent. Position on the graph roughly represents relative relation. Lower panel: summary of characteristics of each tool and suggested decision pipeline to choose the right tool

The aim of this study is to present a benchmark comparison for current clustering methods for mass cytometry data and to help researchers select the suitable tool based on the features of their specific data. To this end, we considered the precision (external comparison), coherence (internal comparison), and stability of different clustering methods. As shown by our results, this comparison procedure comprehensively depicts the characteristics of each tool, providing clear guidance for tool selection (Fig. 5). If researchers wish to determine the pros and cons of other existing or novel tools in the future, this benchmarking framework can be applied to make a thorough comparison.

### Semi-supervised tools fit the task of finding known clusters

As expected, both semi-supervised tools resulted in better performance in terms of precision and stability than unsupervised approaches. This strength was observed in experiments with varying sample sizes, numbers of features, and different indicators (accuracy, *F*-measure, NMI, ARI), suggesting that the advantage of semi-supervised approaches in precision is dominant and exhaustive. Thus, the ability to precisely and robustly reproduce manual labels makes semi-supervised tools suitable for situations where researchers focus on the known cell types with reliable prior knowledge.

The two semi-supervised tools compared in our study, LDA and ACDC, have a fundamental difference in terms of prior input knowledge: LDA requires a training set with manual labels as the input, whereas ACDC requires a “marker × cell type” table that defines the relationship between features and labels. This difference is vital for the choice of semi-supervised tools. In our study, LDA outperformed ACDC in most of the indicators, including precision, stability, and runtime, which made LDA the preferred tool in most conditions. However, LDA depends on a reliable, homogenous training set. When there is no available training set with manual labels, or the training set and test set are heterogeneous (i.e., samples come from different tissues or cell lines, but training set contains only one tissue/cell line), ACDC would be the better choice (Fig. 5 Q2).

Another interesting result is that the performance of semi-supervised tools was beaten by unsupervised tools with respect to coherence (internal evaluation), suggesting that defining cell types based on isolated markers might not precisely capture the inner structure of the data. This observation is not surprising, considering that the number of bi-axal plots required to depict the relationship among features increases exponentially as the number of features increases [12]. Using only dozens of bi-axal plots is thus unlikely to capture the whole picture. The human-subjective judgment of manual gating [34] is another factor that hinders semi-supervised tools from characterizing the objective features of CyTOF data.

### PhenoGraph and FlowSOM are the top-performing unsupervised tools

The manual gating of mass cytometry data requires heavy labor and results in issues regarding reproducibility and subjectivity. Efforts to reduce such burdens have given rise to a wide variety of unsupervised approaches that partition cell populations according to the natural structure of cell data [12]. Our results showed that two outstanding approaches, PhenoGraph and FlowSOM, gave more precise and coherent clustering results than other approaches. Each of these two approaches had an impressive ability to produce coherent clustering results; PhenoGraph showed higher stability, and FlowSOM had the highest precision. We suggest PhenoGraph and FlowSOM as the two top-tier choices when researchers are looking to focus more on the inner structure of the data instead of relying on external prior knowledge.

An apparent limitation of FlowSOM is that it requires the number of clusters to be predetermined (Fig. 5 Q5). Predetermining the number of clusters would be difficult for exploratory experiments, where even a rough estimation of cell-type diversity is hardly available. Although FlowSOM provides an alternative option to automatically estimate the number of clusters within a given range, our results suggest that performing this automatic estimation worsens the performance of FlowSOM. Furthermore, even if a large estimate range (up to twice the number manual labels) was provided, FlowSOM consistently selected a small number of clusters. We believe that the default setting (inputting a predetermined number of clusters) is the optimal setting for FlowSOM, which partially limits the application of this promising tool.

### Sample size has a vital impact

An essential challenge for CyTOF technology is that sample size can vary significantly among different CyTOF experiments [2]. Our results demonstrated that various tools exhibited different performance results when dealing with varying sample sizes; thus, sample size must be taken into consideration when choosing the appropriate tools.

As shown in Fig. 3, the number of clusters found by PhenoGraph and Xshift positively correlated to sample size. This trend could be alleviated, but not eliminated, by the usage of Elbow Plot Determination in Xshift. We reasoned that the impact of large sample size on the number of clusters might have arisen from the inner characteristics of density-based partitioning methods. Generally speaking, both the Louvain method and other modularity maximization algorithms aim to find the optimal partition of a network that reaches a maximum “Newman-Girvan modularity,” or *Q*_max_. However, the maximization of *Q* suffers from the problem of extreme degeneracy: there is typically an exponential number of distinct partitions that are close to the optimum [35]. As the size of the network grows, the number of local optimal solutions grows geometrically, veiling the desired optimal partition. Furthermore, the many locally optimal solutions are often topologically dissimilar [35], which gives rise to inconsistent outputs. This characteristic introduces the potential risk that PhenoGraph and Xshift may be overwhelmed by extremely large sample sizes.

The impact of sample size on flowMeans was inconsistent. In one case, the performance of flowMeans declined when sample size grew large (Fig. 3); in another case, flowMeans frequently introduced the error of singularity and array dimensions in R when the sample size was smaller than 40,000. Although experienced users may modify the source R code to avoid these errors, we believe that this practice is not advisable for common researchers without extensive programming experience in R. Comparatively speaking, FlowSOM had better precision and stability with large sample sizes and is the best alternative choice when dealing with large amounts of cells.

### Clustering resolution varies among different tools

Clustering resolution, the ability to detect small and refined clusters, has seldom been evaluated by previous publications, partly because many parameter settings can impact the number of clusters identified by each tool. By using the default settings for each tool, we found that each tool, as well as their different settings, had a distinct tendency to over- or underestimate the number of clusters. This tendency should not be neglected, given the fact that an over- or underestimation is biologically significant (Fig. 4). Furthermore, the resolution of the manual label is more or less arbitrary and should not be regarded as “golden standard.” Thus, in most cases, the cell type resolution of CyTOF research is determined by the results of the chosen clustering tool.

In the current study, we found that PhenoGraph and Xshift output relatively larger numbers of clusters and split the manual clusters into smaller sub-clusters. On the contrary, DEPECHE grouped similar manual clusters into larger meta-clusters and ignored the subtle differences among them. If researchers wish to focus on the refined subtypes of cells, the appropriate choice would be PhenoGraph or Xshift. If researchers cannot correctly estimate the number of refined clusters they are looking for, even FlowSOM would not be a good choice as PhenoGraph or Xshift, as the small number of clusters found by automatic estimation of FlowSOM is not likely to be “refined” (Fig. 3). If Xshift and PhenoGraph suffer from instability with large sample sizes, an alternative strategy could be a primary application of FlowSOM or DEPECHE to obtain major cell types, followed by detailed sub-clustering on each major type.

## Conclusions

Our study demonstrates that in the field of mass cytometry analysis, LDA best fits the task of precisely reproducing manual clustering labels. PhenoGraph and FlowSOM are the top unsupervised tools because of their high precision, coherence, and stability. PhenoGraph and Xshift can detect a refined subset of major cell types, whereas DEPECHE and FlowSOM tend to group similar cell types into large meta-clusters. Decision guidance has been provided (Fig. 5) as a concluding summary to facilitate the choice of suitable clustering tools based on users’ specific situations.

## Methods

### Clustering tools

A total of seven unsupervised clustering methods (PhenoGraph, Accense, Xshift, FlowSOM, flowMeans, DEPECHE, and kmeans) and two semi-supervised methods (ACDC, LDA) were compared for mass cytometry data in the study (Table 1). Among them, PhenoGraph, Xshift, FlowSOM, and flowMeans are the best-performance tools in a previous comparison of unsupervised tools by Weber and Robinson [25], DEPECHE is a novel clustering tool [19] for cytometry data that had not been analyzed by Weber et al. [25], and LDA and ACDC are top-performance tools in a previous comparison of semi-supervised tools by Abdelaal et al. [11]. kmeans clustering was implemented using a built-in MATLAB kmeans function. The remaining approaches were implemented using the original articles’ suggestions. All tools were freely available for use from the original articles.

In general, we performed each algorithm on arcsinh-transformed data and with default settings. To minimize the influence of inconsistent transformation and scaling methods, we invalidated all transformation and scaling functions for all software (i.e., standardize = FALSE for flowMeans, transformation = NONE and rescale = NONE for Xshift). All the compared tools were performed on a single PC (Intel® Core™ i5-8400 CPU @ 2.80 GHz, a processor with 8.00 GB memory). By default, Xshift was run using standalone.bat with a minimum memory of 1 GB.

### Datasets

We tested the performance of these nine tools on six mass cytometry datasets that served as “benchmarking datasets” (Additional file 1: Table S1). All of these datasets were biologically well characterized with known cell-type annotations. Among them, Levine13dim, Levine32dim, and Samusik01 are well-known benchmarking CyTOF datasets and have already been summarized by Weber and Robinson in their previous comparison [25]. The other three new datasets were summarized as follows:

1. Muscle-resident cells from healthy adult mice [28]. Twenty-five proteins were used for clustering. Eight major cell populations were identified according to provided gating strategies, including Neg/Neg cells that lacked any known cell markers.
2. In vitro cells from three cell lines—HEK293T, MDA-MB-231, and THP-1 [29]. These cell lines were treated by TNFα to induce a cell cycle transformation. Cells at different time points were collected after treatment. Cells were labeled by four phases: G0/G1, G2, S, and M. A total of 35 markers were measured.
3. Our laboratory’s private human colon data [36]. Cells were collected from healthy colon tissue, polyps of a healthy adult, early-stage colon cancer, and late-stage colon cancer. Nineteen protein markers were used for clustering, and 13 manual labels were generated using gating strategies.

### Pre-processing of datasets

First of all, each dataset was filtered to remove annotation incompleteness, doublets, debris, and dead cells. Then, expression levels of all proteins were normalized by the inverse hyperbolic sine function (denoted by arcsinh) with a scale factor of 5:

$$ {\exp}_{\mathrm{normalized}}=\operatorname{arcsinh}\left(\frac{\exp }{5}\right) $$

All nine tools were applied on the filtered and normalized datasets.

Then, we realized that Levine13dim, Levine32dim, and Samusik01 datasets contained unassigned cells or cells with ambiguous annotations (denoted as “NaN” in each .fcs file), which did not belong to any manually gated populations. For this matter, some researchers would like to discard these unassigned cells since these cells were usually low quality cells, intermediate cells, or cells that did not express on some known markers [11, 23]. There were also some researchers who preferred to include these unassigned cells into the clustering [18, 21]. As the existing researches have done, we did the further pre-processing for these three datasets in the following two ways:

1. We discarded unassigned cells or cells with ambiguous annotations and only clustered cells with manually gated annotations into different populations by the compared tools.
2. We executed each compared tools on all cells including unassigned cells or cells with ambiguous annotations, but calculated the evaluation measures using the subset of annotated cells.

By observing the results of both cases (discarding unassigned cells see Tables 2, 3, and 4, including unassigned cells see Additional file 1: Table S4 and S5) separately, it was not difficult to find that the overall ranking order of compared methods was almost the same. However, comparing the results of each method between these two cases, we found that only unstable methods (such as Accense and Xshift) presented obvious changes, and the relatively stable methods basically remained unchanged under our comparison framework (such as DEPECHE and ACDC). Therefore, we mainly discuss the result analysis for datasets excluding unassigned cells, and the results of including unassigned cells are presented in Additional file 1: Table S4 and S5.

For the other three datasets used, each cell had its own annotated labels. Therefore, we directly performed compared tools on all cells. The manually gated annotations were considered to be true cell populations, and the performances of tested tools were measured by computing several evaluation indices between the obtained labels and the manual annotations.

### Subsampling tests

Since different datasets contain different numbers of cells and analysis on large datasets is very time consuming, we randomly subsampled 20,000 and 40,000 cells (5 times each) from each dataset and applied all tools on them. The largest number of subsampling was set at 40,000 because the Samusik01 dataset contains only 53,173 cells with manual annotations. Internal evaluations, external evaluations, stability tests, and further downstream analysis were conducted on these subsampled cells. To further analyze the impact of sample size on the performance of PhenoGraph, Xshift, FlowSOM, and flowMeans, we carried out additional subsampling tests with sample sizes of 5000, 10,000, 60,000, and 80,000 on 2 datasets: Levine32dim and colon. This was because these two datasets have over 100,000 cells and have moderate numbers of manual labels (14 for Levine32dim and 13 for colon).

An exception to this analysis was when the sample size was less than or equal to 20,000, where flowMeans introduced errors of singularity and array dimensions in more than half of the random sampling tests. We inferred that subsampling data without singularity errors might result in bias, so we did not carry out any tests on flowMeans with sample size of less than or equal to 20,000.

### Internal evaluations measure the homogeneity of clustering results

In the current study, we utilized both internal and external evaluations to measure the clustering performance of different approaches. Internal evaluations are based on the hypothesis that an ideal clustering result should have high similarity within each cluster and high heterogeneity between clusters. These evaluations do not require additional “true labels” and analyze the internal characteristics of a clustering result. Such characteristics make them compatible to give a fair comparison between semi-supervised and unsupervised methods. Three internal evaluation methods were adopted in our study:

1. The Xie-Beni index (XB) [32]. We first calculate the pooled within-group sum of squares (WGSS) which measure the dispersion within each cluster as:
  $$ \mathrm{WGSS}={\sum}_k\frac{1}{n_k}\sum \limits_{i<j\in {I}_k}{\left\Vert {M}_i^{\left\{k\right\}}-{M}_j^{\left\{k\right\}}\right\Vert}^2 $$

Where *I*_*k*_ denotes all the samples in cluster *k*, *n*_*k*_ =  ∣ *I*_*k*_∣, and ${M}_i^{\left\{k\right\}}$ represents the observation of sample *i* (for *i* ∈ *I*_*k*_). We then calculate the between-cluster distance as:

$$ {\delta}_1\left(k,{k}^{\prime}\right)=\underset{\begin{array}{c}i\in {I}_k\\ {}j\in {I}_{k^{\prime }}\end{array}}{\min }d\left({M}_i,{M}_j\right) $$

where *d*(*a*, *b*) is the Euclidean distance between *a* and *b*. Based on the above two measurements, XB is defined as:

$$ \mathrm{XB}=\frac{1}{n}\frac{\mathrm{WGSS}}{\underset{k<{k}^{\prime }}{\min }{\delta}_1{\left(k,{k}^{\prime}\right)}^2} $$

2. The Calinski-Harabasz index (CH) [32]. CH also utilizes WGSS to measure the dispersion within each cluster. But unlike XB, CH uses another measurement, between-group sum of squares (BGSS), to measure dispersion between clusters:
  $$ \mathrm{BGSS}=\sum \limits_{i=1}^K{n}_k{\left\Vert {G}^{\left\{k\right\}}-G\right\Vert}^2 $$

where *G*^{*k*}^ denotes the barycenter for cluster *k*, and *G* is the barycenter of all samples. Then, CH is defined as follows:

$$ \mathrm{CH}=\frac{N-K}{K-1}\frac{\mathrm{BGSS}}{\mathrm{WGSS}} $$

3. The Davies-Bouldin index (DB) [32]. DB measures the dispersion within each cluster by average distance to barycenter:
  $$ {\delta}_k=\frac{1}{n_k}\sum \limits_{i\in {I}_k}\left\Vert {M}_i^{\left\{k\right\}}-{G}^{\left\{k\right\}}\right\Vert $$

whereas the dispersion between clusters is measured by:

$$ {\varDelta}_{k{k}^{\prime }}=\left\Vert {G}^{\left\{k\right\}}-{G}^{\left\{{\mathrm{k}}^{\prime}\right\}}\right\Vert $$

Integrating these measures, DB can be written as:

$$ \mathrm{DB}=\frac{1}{K}\sum \limits_{k=1}^K\underset{k^{\prime}\ne k}{\max}\left(\frac{\delta_k+{\delta}_{k^{\prime }}}{\varDelta_{k{k}^{\prime }}}\right) $$

### External evaluations measure the precision of clustering results

On the contrary, external evaluations measure the similarity between a clustering result and the true labels (specifically, manually gated labels in a CyTOF study). External evaluations tend to favor semi-supervised methods over unsupervised methods since they make use of the same true labels.

To measure the precision of predicted clustering, the first step is to obtain a one-to-one mapping between predicted clusters and true cell population. This was achieved by the Hungarian assignment algorithm, a combinatorial optimization algorithm that finds the assignment with the lowest *F*-measure in true cell populations [21]. Then, four different external evaluations were adopted:

1. Single cell-level accuracy (AC) [31], which is defined as the ratio of correctly clustered cells in total cells. Suppose *n* is the total number of cells, *M* is the vector of cluster labels annotated by manual gating, and *T* is the vector of cluster labels predicted by tested approaches. map(*T*_*i*_) is the one-to-one mapping between predicted clusters and actual cell cluster achieved by the Hungarian assignment algorithm. AC is calculated by:
  $$ \mathrm{AC}=\frac{1}{n}\sum \limits_{i=1}^n\delta \left({M}_i,\mathrm{map}\left({T}_i\right)\right) $$

where

$$ \delta \left(x,y\right)=\left\{\begin{array}{c}1, if\ x=y;\\ {}0, if\ x\ne y\end{array}\right. $$

2. Weighted *F*-measure (harmonic mean of precision and recall) [37]. For each cluster *i*, we use
  $$ {F}_i=\frac{2{P}_i{R}_i}{P_i+{R}_i} $$

to calculate its *F*-measure, where ${P}_i=\frac{\mathrm{true}\ \mathrm{positive}}{\mathrm{true}\ \mathrm{positive}+\mathrm{false}\ \mathrm{positive}}$ and ${R}_i=\frac{\mathrm{true}\ \mathrm{positive}}{\mathrm{true}\ \mathrm{positive}+\mathrm{false}\ \mathrm{negative}}$ represent precision and recall of cluster *i*. We summed up the *F*-measure of each cluster over all clusters to obtain the weighted *F*-measure:

$$ F=\sum \frac{n_i}{N}{F}_i $$

where *n*_*i*_ represent the number of cells in cluster *i* and *N* represents the total number of cells.

3. Normalized Mutual Information (NMI) [30]. Suppose *m* ∈ *M* is the clustering assignment from manual gating, *t* ∈ *T* is the clustering assignment from the tested approach, *P*_*M*_(*m*) and *P*_*T*_(*t*) are their probability distributions, and *P*_*MT*_(*m*, *t*) is their joint distribution. Their information entropies are calculated by:
  $$ H(M)=-\sum \limits_m{p}_M(m)\log {P}_M(m) $$

$$ H(T)=-\sum \limits_t{p}_T(t)\log {P}_T(t) $$

We defined mutual information (MI) of *M* and *T* as:

$$ I\left(M,T\right)=\sum \limits_{m,t}{P}_{MT}\left(m,t\right)\log \frac{P_{MT}\left(m,t\right)}{p_M(m){p}_T(t)} $$

If we treat both *M* and *T* as discrete random variables, their statistical redundancy reflects the clustering accuracy (note that a perfect clustering result *T* and the true labels *M* are completely redundant because they contain the same information). *I*(*M*, *T*) captures this redundancy, but its normalized form:

$$ \mathrm{NMI}=\frac{2I\left(M,T\right)}{H(M)+H(T)} $$

is a more commonly used evaluation. The value of NMI would be large if *T* is an optimal clustering result. In an ideal situation, *T* = *M* corresponds to NMI = 1.

4. Adjusted Rand Index (ARI) [38]. Given two different partitions of a same set of samples, *X*_*i*_ (1 ≤ *i* ≤ *r*) and *Y*_*j*_ (1 ≤ *j* ≤ *s*), we denote *n*_*ij*_ as the number of samples that are in both *X*_*i*_ and *Y*_*j*_, *n*_*ij*_ = |*X*_*i*_ ∩ *Y*_*j*_|. Let ${a}_i={\sum}_{j=1}^s{n}_{ij}$ and ${b}_j={\sum}_{i=1}^r{n}_{ij}$, we have ∑*a*_*i*_ =  ∑ *b*_*j*_ =  ∑ *n*_*ij*_ = *n*. We can define ARI as:
  $$ \mathrm{ARI}=\frac{\sum_{ij}\left(\genfrac{}{}{0pt}{}{n_{ij}}{2}\right)-\left[{\sum}_i\left(\genfrac{}{}{0pt}{}{a_i}{2}\right){\sum}_j\left(\genfrac{}{}{0pt}{}{b_j}{2}\right)\right]/\left(\genfrac{}{}{0pt}{}{n}{2}\right)}{\frac{1}{2}\left[{\sum}_i\left(\genfrac{}{}{0pt}{}{a_i}{2}\right)+{\sum}_j\left(\genfrac{}{}{0pt}{}{b_j}{2}\right)\right]-\left[{\sum}_i\left(\genfrac{}{}{0pt}{}{a_i}{2}\right){\sum}_j\left(\genfrac{}{}{0pt}{}{b_j}{2}\right)\right]/\left(\genfrac{}{}{0pt}{}{n}{2}\right)} $$

which measures the similarity between partition *X* and *Y*.

### Evaluation of stability

In this study, we analyzed the stability (robustness) of different tools from two aspects: robustness against random subsampling and robustness against varying sample sizes. The robustness against random subsampling was evaluated using data from subsampling tests with 40,000 cell samples. For any given tool, dataset, and index, there were five values from five subsampling tests. After calculating the standard deviation and mean of these five values, we defined the coefficient of variation (CV) as:

$$ \mathrm{CV}=\frac{\mathrm{Standard}\ \mathrm{Deviation}}{\mathrm{Mean}} $$

For each tool, there were 24 CVs for external evaluation (6 datasets and 4 indices). Their distribution was calculated as a ridge plot (Fig. 2), and we compared the robustness among tools by comparing the median and extreme values of the distribution of CVs.

The evaluation of robustness against varying sample size was conducted similarly, except that CV was replaced by relative difference (RD) between 20,000 and 40,000 cell subsampling tests. For any given tool, dataset, and index, RD was defined as:

$$ \mathrm{RD}=\frac{\left(\mathrm{mea}{\mathrm{n}}_{40k}-\mathrm{mea}{\mathrm{n}}_{20k}\right)}{\mathrm{mea}{\mathrm{n}}_{20k}} $$

### Evaluation of the number of clusters

Among the nine tools we compared, kmeans, FlowSOM, LDA, and ACDC required the number of clusters as an input, flowMeans by default did not require this input, and the remaining tools automatically estimated the number of clusters. To test the stability of each tool, we recorded the number of clusters obtained by flowMeans, PhenoGraph, Accense, Xshift, and DEPECHE in each subsampling test. The standard deviation for each tool was calculated to represent the stability of the tool.

For FlowSOM and Xshift, there are widely applied alternative settings that impacted the number of detected clusters: Elbow Plot Determination to estimate *K* for KNN (Xshift) and automatic estimation of the number of clusters (FlowSOM). We evaluated the performances using these settings, together with PhenoGraph and flowMeans, on the Levine32dim and colon datasets. For FlowSOM, the cluster number estimation range was set at 1 to 2 times the number of manual labels. This range proved to be wide enough given the fact that FlowSOM consistently estimated a relatively low number of clusters.

### Evaluation of clustering resolution

To evaluate the ability of Xshift and PhenoGraph to find refined sub-clusters of manual labels, we defined a many-to-one alignment between predicted clusters and manual labels: if more than half of cells from a predicted cluster belonged to one manual label, we considered this predicted cluster to be a sub-cluster of the corresponding manual label. Under this alignment, we recalculated the *F*-measure, NMI, and ARI. To verify whether Xshift and PhenoGraph can resolve heterogeneity in sample origin in colon data, we defined that one predicted cluster is origin-specific if more than half of its cells come from one sample origin (normal tissue, polyps, early-stage cancer, or late-stage cancer). The fact that most of the predicted clusters can be aligned to one manual label and that this alignment significantly improved precision demonstrates that Xshift and PhenoGraph indeed found the sub-clusters of manual labels. The fact that the majority of Xshift clusters were origin-specific demonstrates that Xshift is capable of resolving heterogeneity of sample origin.

## Supplementary information

**Additional file 1: **Supplementary Method. **Table S1.** Data sets tested in the study. **Table S2.** Impacts of different transformation methods. **Table S3.** Internal evaluation for manual labels. **Table S4.** Summary of external evaluations including unassigned cells. **Table S5**. Summary of internal evaluations including unassigned cells. **Figure S1.** Flowchart of the study. **Figure S2.** Runtime and F-measure of semi-supervised tools (A-C) and unsupervised tools (D-F) on Levine32dim, Cell Cycle and colon data sets. **Figure S3.** Impact of limited training sets on the performance of LDA. **Figure S4.** Stability of each tool evaluated by internal evaluations. **Figure S5.** Evaluation of impacts of sample size on colon data. **Figure S6.** Clustering resolution for PhenoGraph (colon data) and DEPECHE (Levine13dim data). **Figure S7.** Gating strategy for colon data.

**Additional file 2.** Supplementary data.

**Additional file 3.** Review history.

## Data Availability

The Levine13dim, Levine32dim, and Samusik01 datasets are available in the “flowrepository” repository, http://flowrepository.org/id/FR-FCM-ZZPH. The muscle dataset is available at https://community.cytobank.org/cytobank/experiments/81774. The Cell Cycle dataset is available at https://community.cytobank.org/cytobank/experiments/68981. The private colon cancer dataset is available at http://flowrepository.org/id/FR-FCM-Z27K. All codes necessary for the current study are available at https://github.com/WeiCSong/cytofBench [39].
