# Supplementary material for: A comparison framework and guideline of clustering methods for mass cytometry data
Source: Genome Biol. 2019 Dec 23;20:297. doi: 10.1186/s13059-019-1917-7 (PMC6929440; doi:10.1186/s13059-019-1917-7)
Supplement: Supplementary file 1 — Additional file 1: Supplementary Method. Table S1. Data sets tested in the study. Table S2. Impacts of different transformation methods. Table S3. Internal evaluation for manual labels. Table S4. Summary of external evaluations including unassigned cells. Table S5. Summary of internal evaluations including unassigned cells. Figure S1. Flowchart of the study. Figure S2. Runtime and F-measure of semi-supervised tools (A-C) and unsupervised tools (D-F) on Levine32dim, Cell Cycle and colon data sets. Figure S3. Impact of limited training sets on the performance of LDA. Figure S4. Stability of each tool evaluated by internal evaluations. Figure S5. Evaluation of impacts of sample size on colon data. Figure S6. Clustering resolution for PhenoGraph (colon data) and DEPECHE (Levine13dim data). Figure S7. Gating strategy for colon data. [file 13059_2019_1917_MOESM1_ESM.docx]

**Additional file for “A comparison framework and guideline of clustering methods for mass cytometry data”**

**Supplementary Method**

**Generation of private benchmarking data**

The acquisition and detailed description of Colon cancer mass cytometry data can be referred to [1], and the original data in the form of FCS file can accessed from FlowRepository (http://flowrepository.org/) via accession number FR-FCM-Z24L. By removing doublets, dead cells, and cell debris, we classified T cells into 13 cell types as true labels using typical biaxial gating hierarchy strategy, as shown in Figure S7. Considering that the gated data involves more than four million cells, directly sampling data of different scales (ranging from 2,000 to 80,000 cells) from which may change its intrinsic subpopulation structure. In order to keep the relative size of each cell type as consistent as possible with the fact, we randomly sampled data from each cell type at a rate of 3% and simultaneously concatenated many FCS files into a single file as the final data used in this paper. The used colon cancer data can be obtained from FlowRepository (see Data and code availability section in main text), which contains 130,667 cells and 13 subpopulations.

[1] Zhang T, Lv J, Tan Z, et al. Immunocyte profiling using single-cell mass cytometry reveals EpCAM+ CD4+ T cells abnormal in colon cancer[J]. Frontiers in Immunology, 2019, 10: 1571.

**Evaluation of impacts of different transformation methods on performance of clustering tools**

In the current study, we applied five transformation methods to colon data set and ran five top-performance unsupervised tools on these transformed data to evaluate the impact of transformation methods. These methods are:

1) asinh: arcsinh(expression/5), as described in Method of main text.

2) asinh with non-negative value (ANN): This is the suggested methods by Xshift. Before asinh transformation, a specified noise threshold (we set at 1) will be subtracted from every raw value and then all the negative values will be set to zero.

3) asinh with randomized negative value (ARN): This is the suggested methods by Phenograph. ARN is similar to ANN except that negative values are randomized to a normalization distribution rather than set to zero.

4) Logicile (log): The default transformation methods for flow cytometry data analysis. This is implemented in flowCore R package.

5) scale: The default methods of flowMeans. After asinh transformation, values for each marker are scaled to [0,1].

**Table S1 Data sets tested in the study**

| datasets | No. cells | No. manually gated cells | No. clusters | | | No. markers |
| --- | --- | --- | --- | --- | --- | --- |
| Levine13dim | 167,044 | 81,747 | 24 | | 13 | |
| Levine32dim | 265,627 | 104,184 | 14 | | 32 | |
| Samusik01 | 86,864 | 53,173 | 24 | | 39 | |
| muscle | 585,133 | 585,133 | 8 | | 25 | |
| Cell Cycle | 81,594 | 81,594 | | 4 | 35 | |
| colon | 130,667 | 130,667 | 13 | | 19 | |

**Table S2 Impacts of different transformation methods**

| index | method | DEPECHE | FlowSOM | flowMeans | Phenograph | Xshift |
| --- | --- | --- | --- | --- | --- | --- |
| Number  Of cluster | Asinh | 4.4±0.55 | 13 | 14.2±2.68 | 27.0±1.73 | 68.5±1.93 |
|  | ANN | 5.4±0.55 | 13 | 19.6±3.13 | 28.0±2 | 80±3.08 |
|  | ARN | 5.8±0.45 | 13 | 22.4±2.60 | 27.8±2.86 | 80.8±2.86 |
|  | Log | 8.4±1.82 | 13 | NA | 23.8±1.30 | 71.6±3.71 |
|  | scale | 5.0±1.73 | 13 | NA | 25.2±0.84 | 97.2±4.32 |
| Accuracy | Asinh | 0.59±0.02 | 0.56±0.03 | 0.64±0.01 | 0.38±0.02 | 0.31±0.02 |
|  | ANN | 0.60±0.01 | 0.56±0.03 | 0.47±0.06 | 0.38±0.02 | 0.29±0.01 |
|  | ARN | 0.59±0.01 | 0.56±0.05 | 0.46±0.06 | 0.38±0.02 | 0.29±0.01 |
|  | Log | 0.40±0.02 | 0.46±0.01 | NA | 0.31±0.03 | 0.30±0.02 |
|  | scale | 0.44±0.04 | 0.47±0.02 | NA | 0.39±0.01 | 0.24±0.01 |
| F-measure | Asinh | 0.69±0.01 | 0.59±0.02 | 0.68±0.02 | 0.40±0.01 | 0.31±0.01 |
|  | ANN | 0.69±0.02 | 0.60±0.03 | 0.55±0.03 | 0.40±0.02 | 0.27±0.02 |
|  | ARN | 0.67±0.02 | 0.59±0.02 | 0.56±0.03 | 0.40±0.01 | 0.27±0.01 |
|  | Log | 0.45±0.03 | 0.55±0.02 | NA | 0.32±0.02 | 0.30±0.02 |
|  | scale | 0.53±0.03 | 0.56±0.02 | NA | 0.40±0.01 | 0.22±0.01 |
| log_10_(  Calinski-  Harabasz  ) | Asinh | 3.98±0.04 | 3.64±0.02 | 3.60±0.03 | 3.41±0.02 | 3.07±0.01 |
|  | ANN | 3.91±0.03 | 3.63±0.03 | 3.29±0.05 | 3.41±0.03 | 3.01±0.01 |
|  | ARN | 3.89±0.03 | 3.64±0.02 | 3.29±0.08 | 3.42±0.02 | 3.00±0.01 |
|  | Log | 3.73±0.08 | 3.40±0.03 | NA | 3.42±0.02 | 2.89±0.01 |
|  | scale | 3.87±0.08 | 3.34±0.03 | NA | 3.40±0.01 | 2.89±0.01 |

NA indicates that flowMeans introduced singularity error.

**Table S3 Internal evaluation for manual labels**

|  | Calinski-Harabasz | Davies-Bouldin | Xie-Beni |
| --- | --- | --- | --- |
| Levine13dim | 3.815192 | 2.401535 | 1.383491 |
| Levine32dim | 3.8251618 | 1.7089997 | 0.7910288 |
| Samusik01 | 3.6605007 | 1.7133195 | 0.7552018 |
| muscle | 3.755963 | 1.746056 | 1.538259 |
| Cell Cycle | 3.446701 | 2.511195 | 1.049529 |
| colon | 3.471482 | 2.930353 | inf |

Shown are average values in five subsampling tests of 40,000 cells. Calinski-Harabasz and Xie-Beni index shown in log10 scale, as Table3.

**Table S4 Summary of external evaluations** **including unassigned cells**

| **Datasets** | **Methods** | **External evaluations** | | | | |
| --- | --- | --- | --- | --- | --- | --- |
|  |  | **Accuracy** | **F-measure** | | **NMI** | **ARI** |
| Levine13dim | Accense | 0.7801±0.0726 | 0.8210±0.0502 | 0.8589±0.0195 | | 0.8355±0.0563 |
|  | PhenoGraph | 0.8831±0.0026 | 0.8779±0.0038 | 0.8640±0.0024 | | 0.8641±0.0065 |
|  | Xshift | 0.6157±0.0411 | 0.7056±0.0284 | 0.7544±0.0132 | | 0.6250±0.0644 |
|  | kmeans | 0.7597±0.0188 | 0.8009±0.0126 | 0.8178±0.0096 | | 0.7551±0.0306 |
|  | FlowSOM | 0.6878±0.0001 | 0.7809±0.0001 | 0.7219±0.0001 | | 0.6650±0.0001 |
|  | DEPECHE | 0.6223±0.0004 | 0.7624±0.0001 | 0.6208±0.0005 | | 0.6054±0.0004 |
|  | ACDC | 0.9225 | 0.9176 | 0.9159 | | 0.9320 |
| Levine32dim | Accense | 0.6449±0.1071 | 0.7472±0.0749 | 0.8280±0.0382 | | 0.6269±0.1210 |
|  | PhenoGraph | 0.7087±0.0516 | 0.7995±0.0360 | 0.8341±0.0154 | | 0.7025±0.0495 |
|  | Xshift | 0.4822±0.0295 | 0.6298±0.0302 | 0.6925±0.0059 | | 0.4144±0.0326 |
|  | kmeans | 0.5533±0.0662 | 0.6754±0.0527 | 0.7750±0.0187 | | 0.5686±0.0598 |
|  | FlowSOM | 0.7229±0.0001 | 0.7987±0.0001 | 0.7712±0.0000 | | 0.6674±0.0000 |
|  | DEPECHE | 0.8904±0.0000 | 0.9201±0.0000 | 0.8407±0.0000 | | 0.9270±0.0000 |
|  | ACDC | 0.9954 | 0.9955 | 0.9622 | | 0.9885 |
| Samusik01 | Accense | 0.6182±0.0936 | 0.7194±0.0760 | 0.8161±0.0309 | | 0.6233±0.1310 |
|  | PhenoGraph | 0.8831±0.0114 | 0.9034±0.0087 | 0.8679±0.0078 | | 0.8767±0.0228 |
|  | Xshift | 0.6411±0.0956 | 0.7422±0.0693 | 0.7710±0.0278 | | 0.6469±0.0937 |
|  | kmeans | 0.5860±0.0536 | 0.7003±0.0402 | 0.7727±0.0119 | | 0.5711±0.0579 |
|  | FlowSOM | 0.8521±0.0079 | 0.8709±0.0034 | 0.8337±0.0033 | | 0.8216±0.0137 |
|  | DEPECHE | 0.8276±0.0000 | 0.8714±0.0000 | 0.7370±0.0000 | | 0.8144±0.0000 |
|  | ACDC | 0.9688 | 0.9665 | 0.9390 | | 0.9600 |

Data shown as mean±standard deviation. The results of flowMeans was not recorded due to its singularity errors. LDA was only executed on manually annotated cells, so its result was not presented here. ACDC was performed on all cells once.

**Table S5 Summary of internal evaluations including unassigned cells**

| **Datasets** | **Methods** | **Internal evaluations** | | |
| --- | --- | --- | --- | --- |
|  |  | **CH** | **DB** | **XB** |
| Levine13dim | Accense | 3.3846±0.0891 | 1.7698±0.0732 | 1.9003±0.0282 |
|  | PhenoGraph | 3.5909±0.0168 | 1.3988±0.0365 | 1.3355±0.0071 |
|  | Xshift | 3.2166±0.0303 | 2.4775±0.1528 | 1.6051±0.0399 |
|  | kmeans | 3.5299±0.0124 | 1.8782±0.0641 | 1.4113±0.0053 |
|  | FlowSOM | 3.4413±0.0005 | 1.6275±0.0019 | 1.1540±0.0380 |
|  | DEPECHE | 4.0094±0.0017 | 1.0762±0.0125 | 0.9219±0.0177 |
|  | ACDC | 4.2689 | 1.5400 | 1.4863 |
| Levine32dim | Accense | 3.1771±0.0693 | 1.8570±0.2168 | 0.6527±0.0118 |
|  | PhenoGraph | 3.2661±0.0328 | 1.7178±0.0540 | 0.9166±0.0008 |
|  | Xshift | 2.9025±0.0327 | 2.7694±0.0789 | 0.9152±0.0051 |
|  | kmeans | 3.4578±0.0102 | 2.0994±0.1613 | 0.9617±0.0091 |
|  | FlowSOM | 3.4478±0.0005 | 1.4734±0.0044 | 0.6285±0.0008 |
|  | DEPECHE | 3.7371±0.0000 | 1.4799±0.0000 | 0.7658±0.0070 |
|  | ACDC | 3.8724 | 1.4900 | 0.7946 |
| Samusik01 | Accense | 3.2088±0.0439 | 1.9394±0.1417 | 0.7155±0.0009 |
|  | PhenoGraph | 3.4832±0.0274 | 1.5492±0.0440 | 0.7990±0.0108 |
|  | Xshift | 3.1892±0.0484 | 2.5105±0.1915 | 0.9028±0.0043 |
|  | kmeans | 3.5079±0.0053 | 1.8969±0.0787 | 0.8544±0.0028 |
|  | FlowSOM | 3.4651±0.0008 | 1.5351±0.0018 | 0.8250±0.0148 |
|  | DEPECHE | 3.8646±0.0000 | 1.3674±0.0000 | 0.9259±0.0080 |
|  | ACDC | 3.7989 | 1.3676 | 0.7530 |

Data shown as mean ± standard deviation. CH: Calinski-Harabasz index (log10 transformed), DB: Davies-Bouldin index, XB: Xie-Beni index (log10 transformed). ACDC was performed on all cells once.


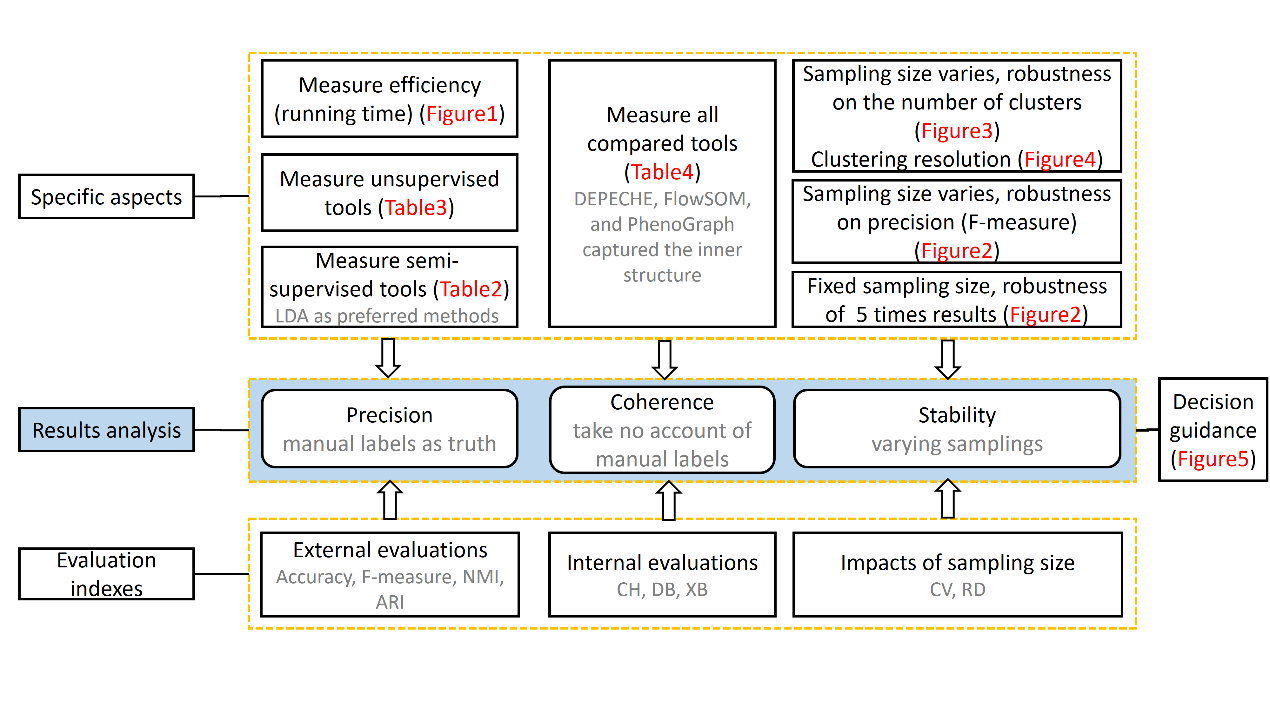


**FigureS1. Flowchart of the study.**

**
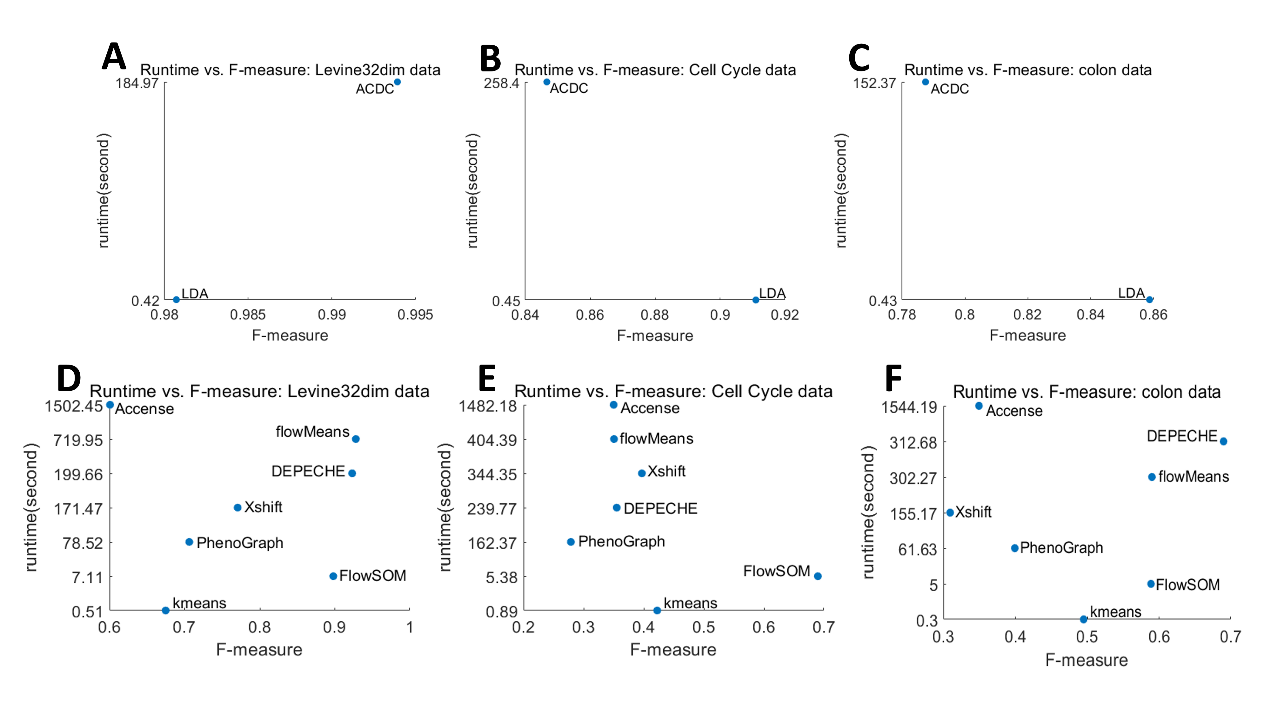
**

**FigureS2. Runtime and F-measure of semi-supervised tools (A-C) and unsupervised tools (D-F) on Levine32dim, Cell Cycle and colon data sets.**

**
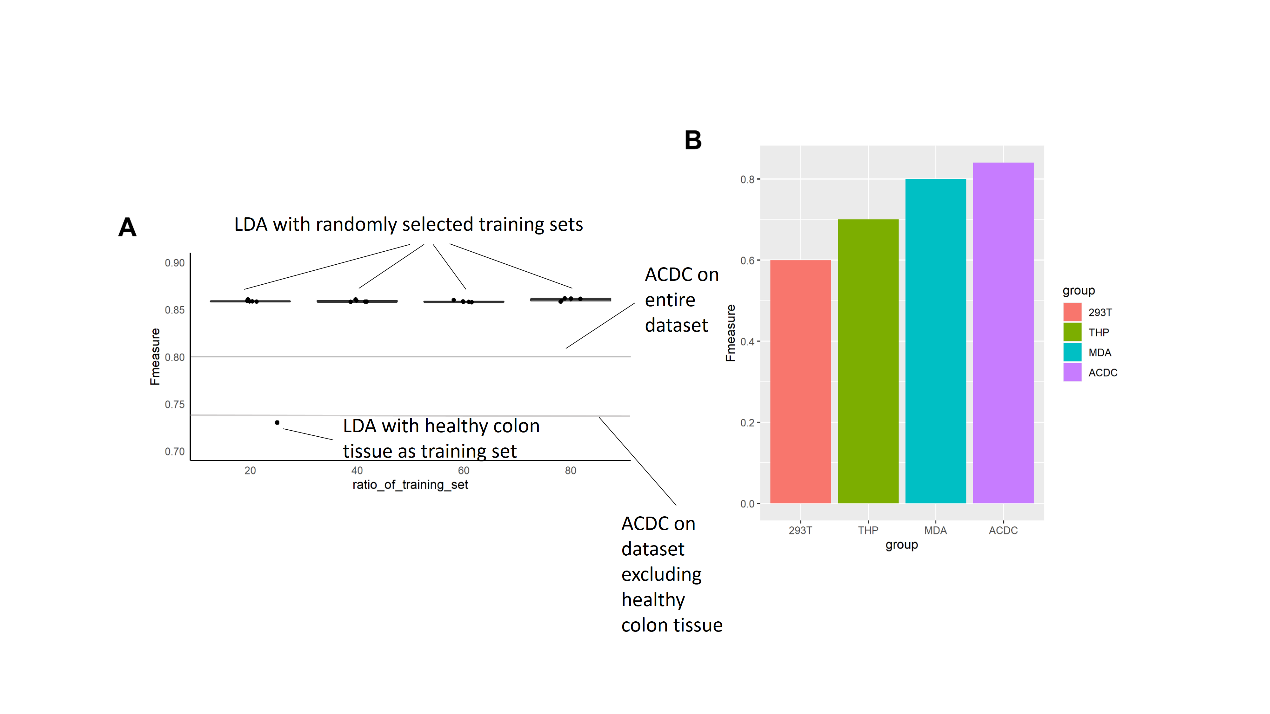
**

**FigureS3. Impact of limited training sets on the performance of LDA.**


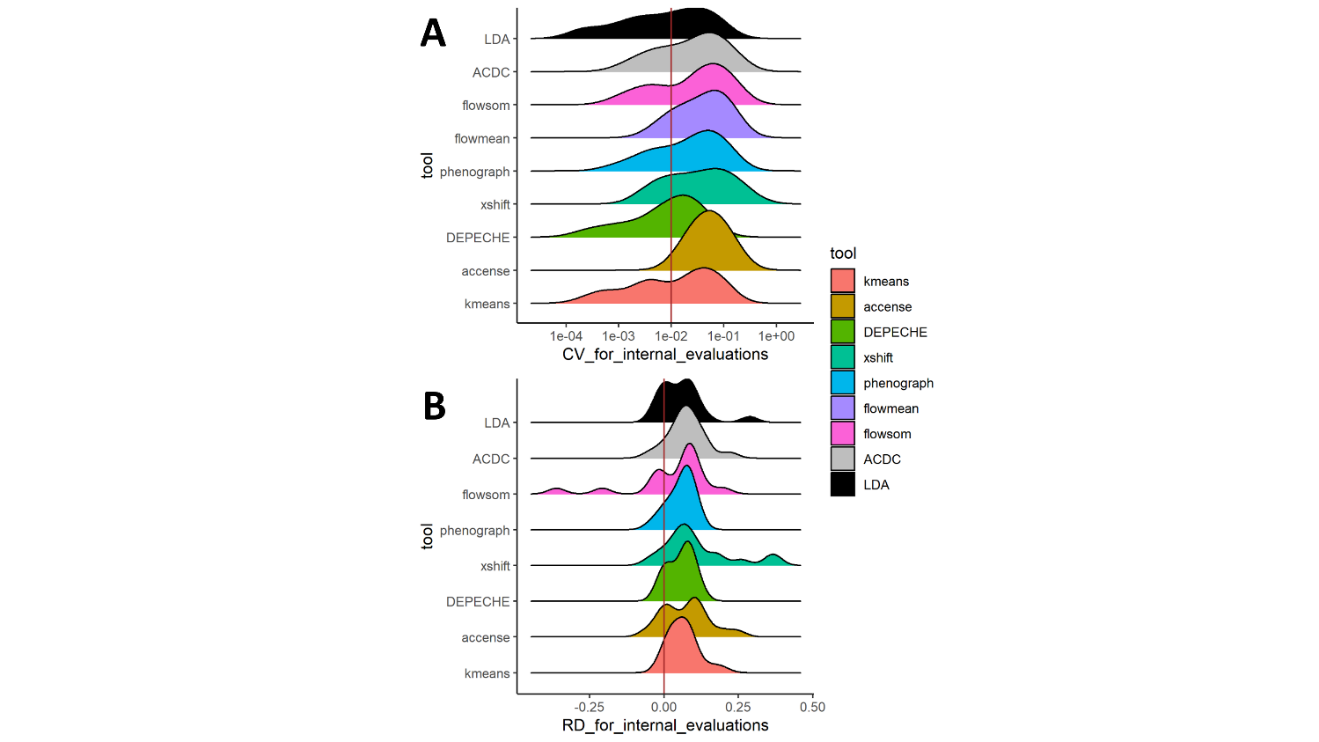


**FigureS4. Stability of each tool evaluated by internal evaluations.**


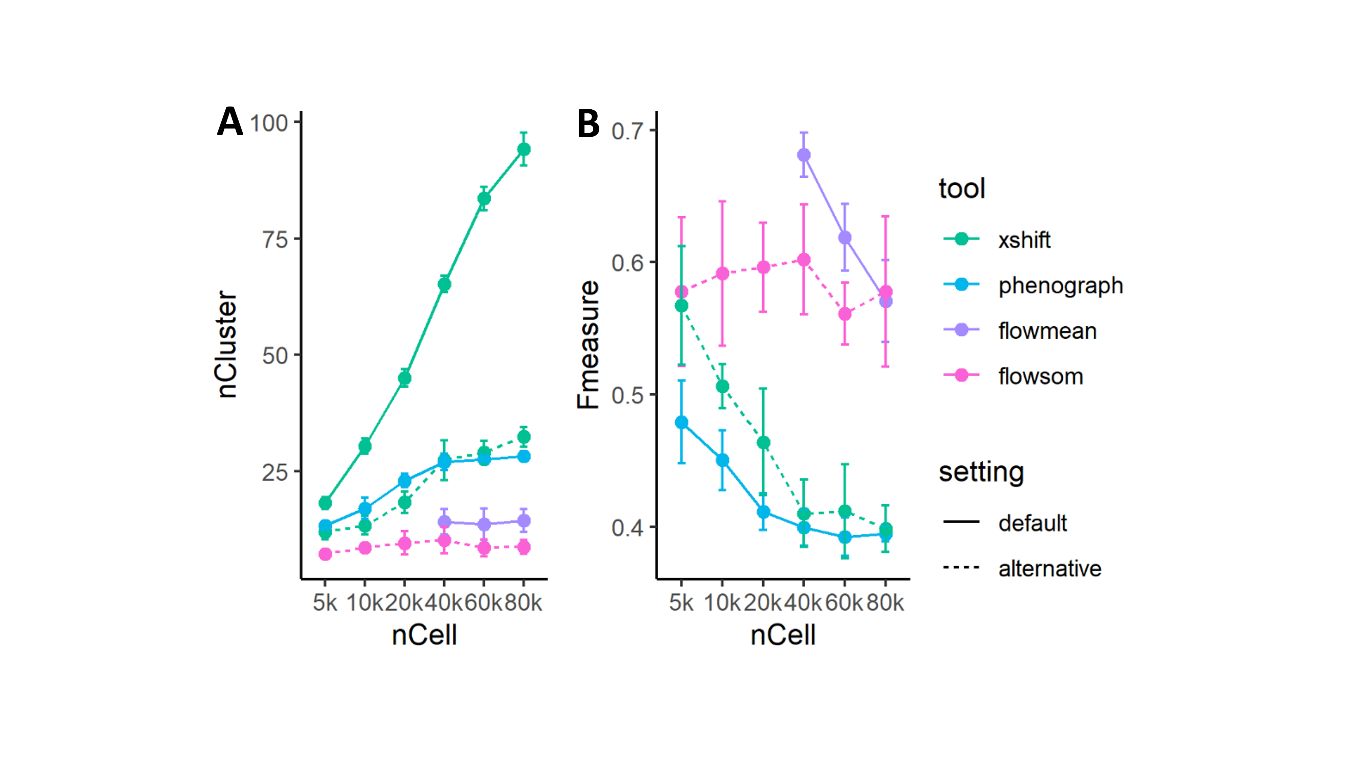


**FigureS5. Evaluation of impacts of sample size on colon data**

**
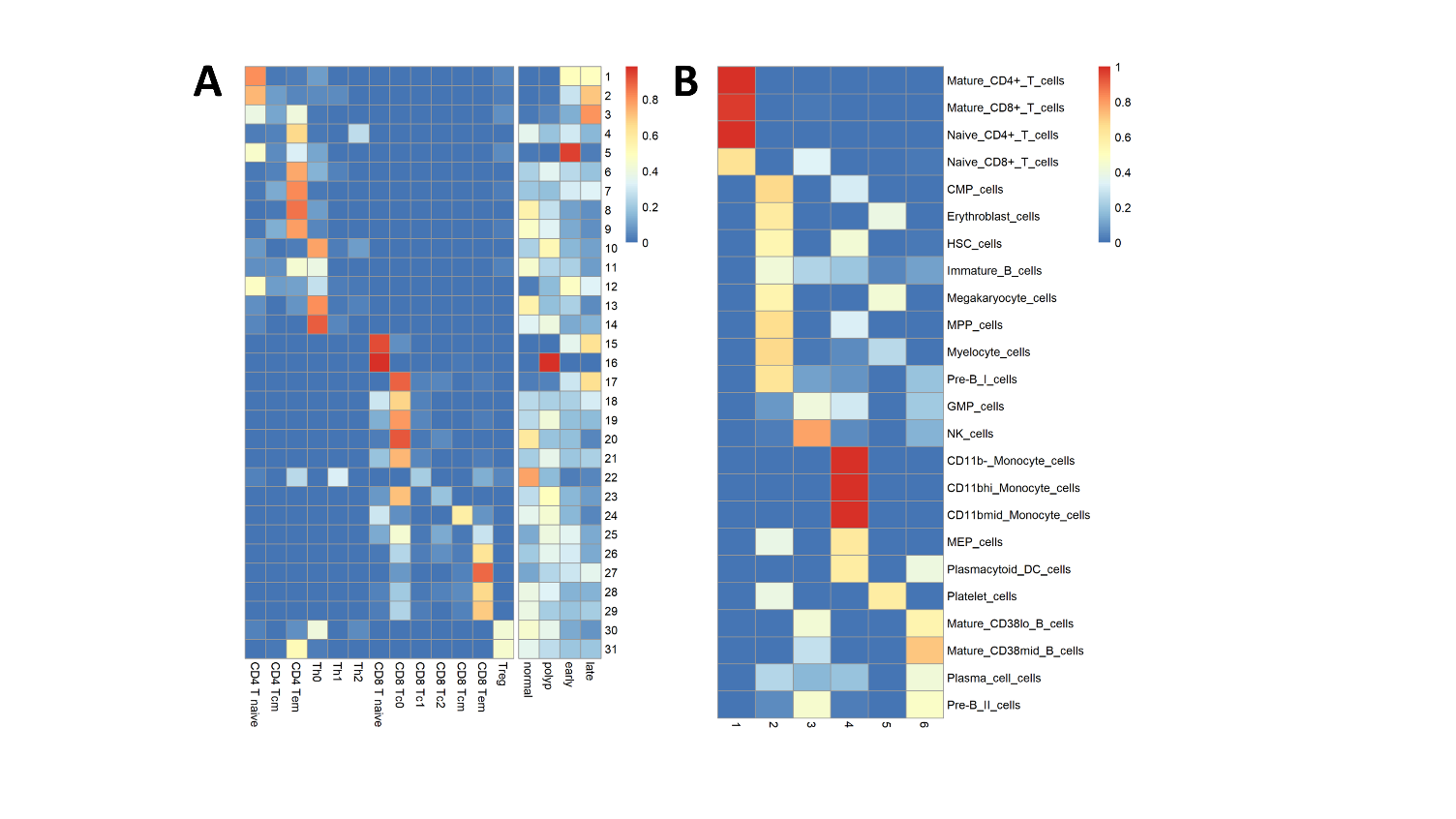
**

**FigureS6. Clustering resolution for PhenoGraph (colon data) and DEPECHE (Levine13dim data)**


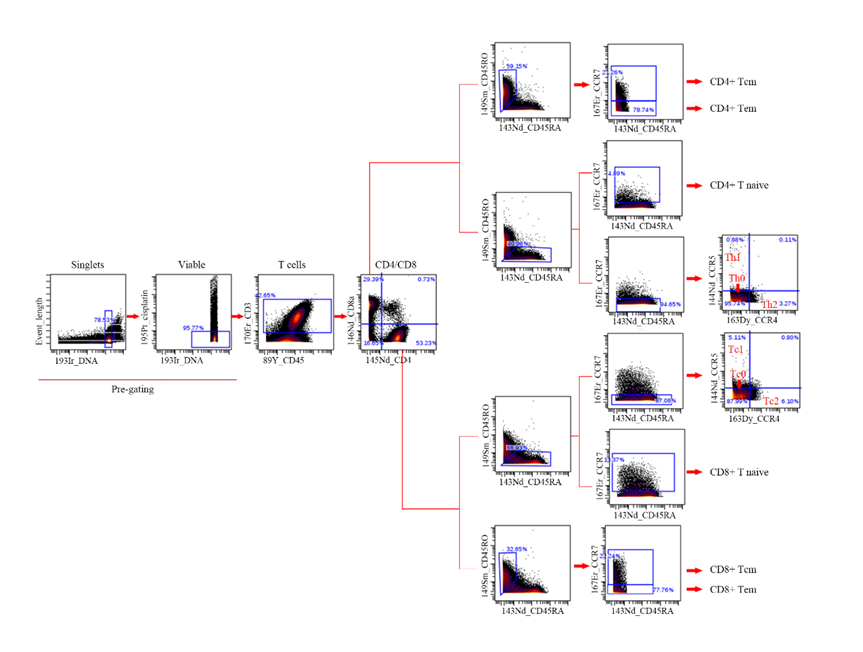


**Figure S7 Gating strategy for colon data**
