# Supplementary material for: A comparison framework and guideline of clustering methods for mass cytometry data
Source: Genome Biol. 2019 Dec 23;20:297. doi: 10.1186/s13059-019-1917-7 (PMC6929440; doi:10.1186/s13059-019-1917-7)
Supplement: Supplementary file 3 — Additional file 3. Review history. [file 13059_2019_1917_MOESM3_ESM.docx]

Review History

**First round of review**

**Reviewer 1**

**Are you able to assess all statistics in the manuscript, including the appropriateness of statistical tests used?**

Yes, and I have assessed the statistics in my report.

**Comments to author:**

Overall comments

Liu et al. have compared the performance of four unsupervised and two supervised methods in terms of reproducing known cluster labels in three publicly available mass cytometry datasets. While the overall aims of (i) comparing unsupervised and supervised analysis strategies and (ii) performing an updated benchmark of available clustering methods are of interest, the paper in its current form does not meet these aims. In particular: comparing unsupervised and supervised methods according to the same evaluation metrics (e.g., F measure) does not provide a meaningful comparison; and the study does not include a comprehensive selection of methods. Specific comments are summarized below.


Major comments

(1) In this study, unsupervised and supervised methods are both evaluated according to the same metrics (e.g., accuracy, F measure, and normalized mutual information for reproducing known cluster labels; see Table 2). Since the supervised methods give higher scores, this is then used to justify the overall conclusion that supervised methods should generally be preferred.

However, comparing unsupervised and supervised methods in this way does not provide a meaningful comparison. It makes sense to compare unsupervised methods against each other, and supervised methods against each other -- but these two types of methods are performing fundamentally different tasks, so it does not make sense to compare between the two groups in this manner (i.e., using the same metrics, such as F measure).

In addition, more discussion should be included about the types of analyses that the two approaches (unsupervised and supervised) are useful for. For example, unsupervised can be used to identify new populations, while supervised is useful when a reliable reference is available. These fundamental differences between the two types of approaches are not clearly explained in the paper; instead, both approaches are simply evaluated in terms of the ability to reproduce known cluster labels.


(2) The second major issue is that the study does not include a comprehensive selection of methods and datasets.

The text notes in several locations that this is a "comprehensive" comparison, and that previous comprehensive comparisons are lacking. However, in its current form, the study is not comprehensive: (i) It includes only four unsupervised and two supervised methods. By contrast, a previous published benchmark of unsupervised methods included 18 methods (Weber and Robinson, 2016), and a previous benchmark of supervised methods included 4 methods (Abdelaal et al., 2019). All methods included in this study were already included in these previous benchmarks, and no justification is provided for the inclusion of these methods and exclusion of others. (ii) It does not include some of the top-performing methods from previous studies (e.g., FlowSOM and flowMeans from Weber and Robinson, 2016). (iii) It does not include any new methods that were not yet available at the time of the previous benchmarks. (iv) It includes only three datasets, all of which were previously used in the benchmarks by Weber and Robinson (2016) and Abdelaal et al. (2019).


(3) The datasets used in this study contain cell population labels for around half of all cells, with the remaining cells designated as "unassigned". In this study, the authors have excluded the unassigned cells -- so all methods are run on the labeled cells only (see Methods). This creates a much easier clustering (unsupervised) or classification (supervised) task, since the unassigned cells would be exactly the cells where the clustering or classification task is more difficult. This design choice should either be clearly justified in the text, or changed to include all cells (which would likely significantly affect performance for all methods).

A related concern is that it is possible that the "true" cluster labels may be incorrect for some cells in some datasets. This issue should be discussed in the text, in the context of both unsupervised and supervised methods.


(4) The analysis regarding the number of clusters (e.g., Table 3) does not discuss the effects of parameter choices: e.g., for some methods, the number of clusters can easily be controlled by adjusting parameters.

Similarly, the "true" number of clusters in the original datasets depends on the choices made by the authors of the datasets. By using a different clustering resolution (i.e., larger or smaller clusters), it would be possible to define different "true" numbers of clusters in these datasets.


(5) Runtimes and computational efficiency are not discussed. For a comprehensive comparison of methods, this is crucial information that should be included.


(6) Code is not provided. Ideally, a code repository (e.g., GitHub) containing code to reproduce all results and figures in the paper should be provided, to ensure reproducibility of the results.


Minor comments

Additional minor comments:

*     Background (lines 34-35): mentions that "CyTOF achieves a much higher throughput (up to millions of cells vs. thousands of cells)" compared to single-cell RNA-sequencing. This is not strictly true, since recent scRNA-seq studies have now also reached more than a million cells (e.g., Cao et al., 2019: https://www.ncbi.nlm.nih.gov/pubmed/30787437).

*     Background (line 36): mentions "millions of cell subtypes". There is no current evidence that there are this many cell subtypes.

*     Background (line 77): mentions that the study by Weber and Robinson (2016) evaluated clustering accuracy but not stability. This is not correct: this study also evaluated stability (although the main focus was on clustering performance and runtimes).

*     Results (line 115): these values (for accuracy, F measure, and normalized mutual information) do not appear to be shown in the corresponding table (Table 2).

*     Discussion (line 257): this sentence could be interpreted to mean that Abdelaal et al. (2019) are the original authors of the LDA algorithm; this should be re-worded (since the original LDA algorithm has existed for decades).

**Reviewer 2**

**Are you able to assess all statistics in the manuscript, including the appropriateness of statistical tests used?**

Yes, and I have assessed the statistics in my report.

**Comments to author:**

Liu et al. tried to perform a benchmark comparison study of clustering methods for mass cytometry data and offer a guideline for the selection of clustering methods. The intention is good. However, the investigation is not thorough enough, and therefore it can't be accepted in its current status.  I have some comments as below:
Major:
It is a self-proof question that the semi-supervised methods will outperform un-supervised methods since you have more domain prior knowledge to assist clustering. It spoils the manuscript if you list this finding as your top conclusion.
There are so many clustering methods out there. The authors compared too few methods.
The review of the methods is not very precise.  For example, what does "manual curated knowledge" exactly refer to?
About clustering result measurement: Page 18, please specify what is precision and recall. Are they related with map(Ti)?  There is a straightforward metric to measure the similarity of clustering results called adjusted rand index. The authors can consider using it too.
Did the authors consider comparing the running time of these methods?
Minor:
Page 3, line 36, "millions of cell subtypes", is this a reasonable magnitude?
Grammar mistakes, e.g., absent VS. absence, hypothesis VS. hypothesize. Please check thoroughly.

**Authors Response**

**Point-by-point responses to the reviewers’ comments:**

Reviewer #1: Overall comments

Liu et al. have compared the performance of four unsupervised and two supervised methods in terms of reproducing known cluster labels in three publicly available mass cytometry datasets. While the overall aims of (i) comparing unsupervised and supervised analysis strategies and (ii) performing an updated benchmark of available clustering methods are of interest, the paper in its current form does not meet these aims. In particular: comparing unsupervised and supervised methods according to the same evaluation metrics (e.g., F measure) does not provide a meaningful comparison; and the study does not include a comprehensive selection of methods. Specific comments are summarized below.

Major comments

(1) In this study, unsupervised and supervised methods are both evaluated according to the same metrics (e.g., accuracy, F measure, and normalized mutual information for reproducing known cluster labels; see Table 2). Since the supervised methods give higher scores, this is then used to justify the overall conclusion that supervised methods should generally be preferred.

However, comparing unsupervised and supervised methods in this way does not provide a meaningful comparison. It makes sense to compare unsupervised methods against each other, and supervised methods against each other -- but these two types of methods are performing fundamentally different tasks, so it does not make sense to compare between the two groups in this manner (i.e., using the same metrics, such as F measure).

In addition, more discussion should be included about the types of analyses that the two approaches (unsupervised and supervised) are useful for. For example, unsupervised can be used to identify new populations, while supervised is useful when a reliable reference is available. These fundamental differences between the two types of approaches are not clearly explained in the paper; instead, both approaches are simply evaluated in terms of the ability to reproduce known cluster labels.

*Response to Major comment 1: We would like to thank the reviewer for the insightful comments and suggestions. To address these concerns, we separated the comments into two major parts and addressed them sequentially as follows:*

“However, comparing unsupervised and supervised methods in this way does not provide a meaningful comparison. It makes sense to compare unsupervised methods against each other, and supervised methods against each other -- but these two types of methods are performing fundamentally different tasks, so it does not make sense to compare between the two groups in this manner (i.e., using the same metrics, such as F measure).”

*We thank the reviewer for the discussion of direct comparison between unsupervised and semi-supervised methods. In this revised manuscript, we have compared the performance of different tools from three aspects with a total of ten indicators: precision (external evaluation indicators: accuracy, F-measure, Normalized Mutual Information and Adjusted Rand Index), coherence (internal evaluation indicators: Calinski-Harabasz index, Xie-Bini index, Davies-Bouldin index), and stability (Coefficient of variation, relative difference and the number of identified clusters). The runtime of each tool was also discussed. We agreed that it was unfair to compare unsupervised tools to semi-supervised tools in terms of precision, so the analysis of external evaluation was conducted separately within the two categories (see manuscript pages 7-10, lines 128-179). On the other hand, given that internal evaluation is defined on the basis of maximizing both the within-group similarity and between-group dissimilarity, and it simply measures the quality of the clustering result itself without taking external manual labels into account (thus we call it “internal”), we infer that these internal indicators are capable of comparing the two method categories in an unbiased manner. Thus, we compared the internal evaluations of all tools together to see which tool best captures the inner structure of CyTOF data (Table I & third section of RESULTS corresponding to manuscript pages 10-11, lines 181-219). Another advantage of internal evaluation is that it avoids the arbitrariness of manual labels, as the reviewer mentioned in comment (3).*

*Similarly, the entire framework of discussion was modified. We no longer recommended any unsupervised or semi-supervised tool solely based on any particular indicator; instead, we discussed their respective characteristics in multiple aspects (related to point 2, as shown below). We provide a decision pipeline at the end of discussion to facilitate the users choose the most suitable tool based on their research aim and data characteristics (Figure I).*

**Table I (Same as Table 3 in the manuscript)
Summary of internal evaluations**

| **Datasets** | **Methods** |  | **Internal evaluations** |  |
| --- | --- | --- | --- | --- |
|  |  | **CH** | **DB** | **XB** |
|  | Accense |  |  |  |
|  |  | 3.2409±0.2069 | 2.4746±0.2563 | 0.6473±0.0750 |
|  | PhenoGraph |  |  |  |
|  |  | 3.4250±0.0350 | 2.5692±0.1503 | 0.7864±0.0392 |
|  | Xshift |  |  |  |
|  | kmeans | 3.5620±0.0443 | 1.7337±0.1618 | 0.9131±0.3066 |
|  | flowMeans | 3.9414±0.0011 | 1.5971±0.0013 | 0.7705±0.0165 |
| Cell Cycle |  | 3.5992±0.0283 | 1.6237±0.0341 | 0.7294±0.0393 |
|  | FlowSOM |  |  |  |
|  |  | 3.6294±0.0036 | 1.3578±0.0351 | 0.6669±0.0269 |
|  | DEPECHE |  |  |  |
|  |  | 3.8372±0.0283 | 1.7767±0.0322 | 0.7695±0.0177 |
|  | ACDC |  |  |  |
|  |  | 3.4645±0.0901 | 2.0773±0.3156 | 0.9395±0.0428 |
|  | LDA |  |  |  |
|  |  | 3.4581±0.0036 | 2.5080±0.0191 | 1.0021±0.0358 |
|  | Accense |  |  |  |
|  |  | 3.2688±0.0622 | 1.7064±0.0656 | 1.0427±0.0545 |
|  | PhenoGraph |  |  |  |
|  |  | 3.4231±0.0194 | 1.9030±0.0245 | 1.3997±0.1199 |
|  | Xshift |  |  |  |
|  | kmeans | 3.0706±0.0179 | 2.3218±0.0595 | 1.3207±0.0511 |
|  | flowMeans | 3.5109±0.0079 | 1.9037±0.0501 | 1.4570±0.1055 |
| Colon |  | 3.4793±0.1068 | 1.5864±0.0421 | 1.6706±0.1958 |
|  | FlowSOM |  |  |  |
|  |  | 3.6435±0.0117 | 1.6583±0.0514 | 1.5910±0.1568 |
|  | DEPECHE |  |  |  |
|  |  | 3.9819±0.0375 | 1.7725±0.0250 | 1.6325±0.0930 |
|  | ACDC |  |  |  |
|  |  | 3.5898±0.0440 | 2.0607±0.1160 | 1.4239±0.0754 |
|  | LDA |  |  |  |
|  |  | 3.5100±0.0009 | 2.1773±0.0065 | 1.5564±0.0724 |
|  | Accense |  |  |  |
|  |  | 3.4230±0.0932 | 1.8832±0.1408 | 1.2321±0.0278 |
| Levine13dim |  |  |  |  |
|  | PhenoGraph |  |  |  |
|  |  | 4.0739±0.0176 | 1.4645±0.0346 | 1.3972±0.1349 |

| **Datasets** | **Methods** |  | **Internal evaluations** |  |
| --- | --- | --- | --- | --- |
|  |  | **CH** | **DB** | **XB** |
|  | Xshift |  |  |  |
|  | kmeans | 3.5106±0.0289 | 2.4284±0.0443 | 1.7868±0.0476 |
|  | flowMeans | 3.8508±0.0150 | 2.1550±0.0546 | 1.6213±0.1471 |
|  |  | 4.0475±0.0194 | 1.5030±0.0849 | 1.4234±0.1182 |
|  | FlowSOM |  |  |  |
|  |  | 3.8486±0.0071 | 1.7564±0.0615 | 1.5043±0.1531 |
|  | DEPECHE |  |  |  |
|  |  | 4.2783±0.0174 | 1.1677±0.0342 | 1.3562±0.0392 |
|  | ACDC |  |  |  |
|  |  | 3.9638±0.0110 | 1.4916±0.0370 | 1.3109±0.0948 |
|  | LDA |  |  |  |
|  |  | 3.8288±0.0106 | 2.0046±0.0493 | 1.3828±0.1167 |
|  | Accense |  |  |  |
|  |  | 3.4621±0.0901 | 2.3414±0.0925 | 0.7891±0.0950 |
|  | PhenoGraph |  |  |  |
|  |  | 3.7401±0.0081 | 1.8293±0.0810 | 1.0009±0.0479 |
|  | Xshift |  |  |  |
|  | kmeans | 3.6669±0.0102 | 2.2576±0.1324 | 0.8295±0.1382 |
|  | flowMeans | 3.8761±0.0166 | 2.0587±0.0386 | 0.9972±0.0441 |
| Levine32dim |  | 3.8546±0.0393 | 1.6975±0.2199 | 0.7985±0.0709 |
|  | FlowSOM |  |  |  |
|  |  | 3.8244±0.0285 | 1.5974±0.0863 | 0.8366±0.0792 |
|  | DEPECHE |  |  |  |
|  |  | 4.1480±0.0009 | 1.4727±0.0023 | 0.7575±0.0351 |
|  | ACDC |  |  |  |
|  |  | 3.6169±0.0046 | 1.3974±0.0049 | 0.7693±0.1310 |
|  | LDA |  |  |  |
|  |  | 3.8297±0.0007 | 1.7011±0.0099 | 0.7155±0.0139 |
|  | Accense |  |  |  |
|  |  | 3.2254±0.1688 | 2.3190±0.3178 | 0.8420±0.1211 |
|  | PhenoGraph |  |  |  |
|  |  | 3.6052±0.0132 | 1.8619±0.0417 | 1.7228±0.2389 |
|  | Xshift |  |  |  |
| Muscle | kmeans | 3.2898±0.0274 | 2.2460±0.1524 | 2.1455±0.1599 |
|  | flowMeans | 3.9722±0.0022 | 1.7729±0.0099 | 1.4987±0.1212 |
|  |  | 3.3809±0.0565 | 1.7685±0.0769 | 1.3750±0.1428 |

| **Datasets** | **Methods** |  | **Internal evaluations** |  |
| --- | --- | --- | --- | --- |
|  |  | **CH** | **DB** | **XB** |
|  | FlowSOM |  |  |  |
|  |  | 3.8262±0.0146 | 1.4439±0.0462 | 1.3586±0.1473 |
|  | DEPECHE |  |  |  |
|  |  | 4.2639±0.0044 | 1.2235±0.0080 | 1.2892±0.0269 |
|  | ACDC |  |  |  |
|  |  | 3.6900±0.0305 | 1.7186±0.0515 | 1.5641±0.15555 |
|  | LDA |  |  |  |
|  |  | 3.8073±0.0031 | 1.6641±0.0206 | 1.6003±0.0900 |
|  | Accense |  |  |  |
|  |  | 3.2952±0.0476 | 2.3424±0.1454 | 0.6500±0.0266 |
|  | PhenoGraph |  |  |  |
|  |  | 3.7380±0.0033 | 1.4607±0.0424 | 0.7669±0.0831 |
|  | Xshift |  |  |  |
|  | kmeans | 3.2660±0.0134 | 2.7855±0.4644 | 1.2670±0.1654 |
|  | flowMeans | 3.7087±0.0135 | 2.2072±0.0854 | 0.9365±0.0792 |
| Samusik01 |  | 3.4029±0.0381 | 1.6069±0.2374 | 0.6309±0.0475 |
|  | FlowSOM |  |  |  |
|  |  | 3.6941±0.0257 | 1.8967±0.3799 | 0.8286±0.0832 |
|  | DEPECHE |  |  |  |
|  |  | 4.1028±0.0021 | 1.4867±0.0054 | 0.9141±0.0109 |
|  | ACDC |  |  |  |
|  |  | 3.6827±0.0178 | 1.3871±0.0132 | 0.7273±0.0573 |
|  | LDA |  |  |  |
|  |  | 3.6767±0.0080 | 1.6325±0.0242 | 1.0414±0.0808 |

Data shown as mean ± standard deviation. CH: Calinski-Harabasz index (log10 transformed), DB: Davies-Bouldin index, XB: Xie-Beni index (log10 transformed).

**Figure I (Same as Fig. 5 in the manuscript).** Upper panel: relative advantages of each tool. Note, precision and coherence are not exclusive; some tools like FlowSOM and PhenoGraph are both precise and coherent. Position on the graph roughly represents relative relation. Lower panel: summary of characteristics of each tool and suggested decision pipeline to choose the right tool.

“In addition, more discussion should be included about the types of analyses that the two approaches (unsupervised and supervised) are useful for. For example, unsupervised can be used to identify new populations, while supervised is useful when a reliable reference is available. These fundamental differences between the two types of approaches are not clearly explained in the paper; instead, both approaches are simply evaluated in terms of the ability to reproduce known cluster labels.”

*We greatly appreciate the reviewer for this suggestion. As the reviewer pointed out, different algorithms aim to perform different tasks. Specifically, unsupervised tools can work well to discover unknown clusters. We verified this claim by showing that Xshift and PhenoGraph could find sub-clusters with biological significance that were not defined by manual gating (see manuscript pages 16-17 and lines 306-343, Figure II-1 & Figure II-2). Meanwhile, we also discussed that semi-supervised tool is more effective when reliable priori information is available from two points:*

*1) If we used the internal evaluations to measure the performance of ACDC and LDA methods, we found that there are no significant improvements of the two semi-supervised methods over the unsupervised methods. This is because the calculation of internal evaluations has nothing to do with the external manual labels, and they just faithfully measure whether the identified clustering is of well within-group similarity and between-group dissimilarity. On this occasion, manual gating labels are no longer regarded as reliable priori information (see manuscript page 11, lines 211-217).*

*2) LDA depends on a reliable, homogeneous training set. If we selected training set
from a specific single cell line (Cell cycle data) or tissue (colon data), the performance of LDA has declining trend over the performance of training set coming from different cell lines or tissues (see manuscript pages 8-9 and lines 150-161, Figure III).*

*This capability, together with other characteristics found in our study, was integrated into a single decision pipeline (Figure I) which could help researchers decide the tool that best fits the experiment’s circumstances.*

**Figure II-1 (Same as Fig. 4 in the manuscript)** Clustering resolution of Xshift and DEPECHE. A: Each row represents one cluster detected by Xshift; each column represents a manual label (left panel) of tissue origin (right panel). Color of each grid denotes the proportion of cells of that cluster belonging to the corresponding manual label (left) of tissue (right). Thus, row sums of both panels are 100%. Black grids highlight the specific patterns resolved by Xshift. B: Similar to A, but for the relationship between DEPECHE clusters (column) and manual labels of Samusik01 data (row).

**Figure II-2 (Same as Fig. S5 in the manuscript)** Clustering resolution for PhenoGraph (colon data) and DEPECHE (Levine13dim data).

**Figure III (Same as Fig. S2 in the manuscript)** Impact of limited training sets on the performance of LDA.

(2) The second major issue is that the study does not include a comprehensive selection of methods and datasets.

The text notes in several locations that this is a "comprehensive" comparison, and that previous comprehensive comparisons are lacking. However, in its current form, the study is not comprehensive: (i) It includes only four unsupervised and two supervised methods. By contrast, a previous published benchmark of unsupervised methods included 18 methods (Weber and Robinson, 2016), and a previous benchmark of supervised methods included 4 methods (Abdelaal et al., 2019). All methods included in this study were already included in these previous benchmarks, and no justification is provided for the inclusion of these methods and exclusion of others. (ii) It does not include some of the top-performing methods from previous studies (e.g., FlowSOM and flowMeans from Weber and Robinson, 2016). (iii) It does not include any new methods that were not yet available at the time of the previous benchmarks. (iv) It includes only three datasets, all of which were previously used in the benchmarks by Weber and Robinson (2016) and Abdelaal et al. (2019).

*Response to Major comment 2: We thank the reviewer for the comment on the selection of tools and data sets. We have expanded our inclusion of both examined tools and data sets as discussed in below:*

“It includes only four unsupervised and two supervised methods.”

“It does not include some of the top-performing methods from previous studies (e.g., FlowSOM and flowMeans from Weber and Robinson, 2016).”

“It does not include any new methods that were not yet available at the time of the previous benchmarks.”

*In the revised manuscript, a total of nine tools were included: Accense, DEPECHE, FlowSOM, flowMeans, kmeans, PhenoGraph, Xshift, ACDC, and LDA (see Table II and the first section of Clustering tools in METHODS in the manuscript, lines from 459-476 in the manuscript). Among them, PhenoGraph, Xshift, flowSOM and flowMeans were considered as the best-performing tools in a previous comparison by Weber and Robinson (2016), DEPECHE is a novel unsupervised clustering tools for cytometry data that was not previously analyzed by their benchmark, and LDA and ACDC were top-performing tools in another previous comparison of semi-supervised tools by Abdelaal et al. (2019). We believe that the previous benchmarking comparisons by Weber & Robinson and Abdelaal et al. have highlighted the outstanding tools of its kind (semi- or unsupervised) and narrowed the range of tools to choose from. From these efforts, we now have a great opportunity to give a focused and detailed comparison of these tools based on the specific application scenarios that researchers may commonly face. In addition, two moderate tools, kmeans and Accense (density-based), were included to analyze the potential bias between density-based and kmeans-based tools. Note: one of our innovations in this study is that we not only analyze the coherence as internal evaluations (CH, DB and XB) to capture the inner structure of dataset, but also explore the possible relation between internal evaluation indicators and the basic principle of methods. For example, CH and DB naturally favor kmeans-based methods while XB favor density-based methods. However, these facts were not discussed in the previous publications.*

**Table II (Same as Table 1 in the manuscript) Methods compared in the study.**

| Methods | | Implementation tools | Description | ref |
| --- | --- | --- | --- | --- |
| **unsupervised** | Accense | MATLAB | tSNE dimension reduction & 2D projection  Kernel-based estimation of density  Density-based peak-finding and partitioning | [12] |
|  | PhenoGraph | R (cytofkit package) | Detection of k-nearest neighbors of each cell  Jaccard similarity coefficient as connectivity  Community detection based on connection density | [15] |
|  | Xshift | Vortex | Weighted k-nearest neighbor density estimation  Detection of density centroids  Cells linked to centroid via density-ascending paths | [18] |
|  | FlowSOM | R | Self-organizing map (SOM) trained on scaled data  Nodes of SOM connected by minimal spanning tree  Consensus hierarchical meta-clustering of nodes | [37] |
|  | flowMeans | R | K estimated by peak numbers of kernel density  kmeans clustering of estimated K  Merging clusters by distance metrics | [26] |
|  | DEPECHE | R | Tuning penalty by resampling data set  Penalized kmeans clustering | [32] |
|  | kmeans | MATLAB | Standard kmeans procedure |  |
| **semi-supervised** | ACDC | Python | Marker×cell matrix & cell type×marker table  Detect landmark points by community detection  Link cells to landmarks by random walkers | [10] |
|  | LDA | MATLAB | Linear discriminant analysis with training data sets | [8] |

4: “It includes only three datasets, all of which were previously used in the benchmarks by Weber and Robinson (2016) and Abdelaal et al. (2019).”

*We included three additional novel datasets in our comparison: muscle, Cell Cycle, and colon (METHODS, in the section of Datasets, Table III, manuscript page 24 and lines 478-494).*

*A. Muscle-resident cells from normal adult mice (Giordani et al., 2019). 25 proteins were used for clustering. 8 major cell populations were identified following provided gating strategy, including Neg/Neg cells that lacked any known cell markers (manuscript lines 484-486).*

*B. Cell Cycle data from in vitro cells of three cell lines: HEK293T, MDA-MB-231, and THP-1 (Rapsomaniki et al., 2018). These cell lines were treated by TNFα to induce a cell cycle transformation. Cells at different time points after treatment were collected. Cells were labeled by four phases: G0/G1, G2, S and M. A total of 35 markers were measured (manuscript lines 487-490).*

*C. Our laboratory’s human colon data: cells from healthy colon tissue, polyp of healthy adult, early stage colon cancer, and late stage colon cancer. There were 19 protein markers and 13 manual labels (Zhang et al., 2019, manuscript lines 491-494).*

*Compared to the previously included data sets, these new data have some distinct characteristics that are useful for testing the performance of clustering tools:*

*A. They have relatively small numbers of manual labels, which helps us better evaluate the relation between the arbitrariness of manual gating and the performance of unsupervised tools. An extreme example is Cell Cycle data, which has only four labels.*

*B. They come from solid tissue (muscle), solid tumors (colon cancer), and in vitro cell curations (Cell Cycle). Together with previous data sets that come from bone marrow, our comparison roughly reflects the performance of each tool on different tissue types.*

*C. Cell Cycle and colon cancer data contain heterogenous samples (cells from different cell lines or tissue types). As shown by our results (Figure III), the heterogeneity of data significantly influenced the performance of several tools. Therefore, the robustness of a tool against the heterogeneity of the original sample is another comparison in our analysis, which has seldom been mentioned in previous literature.*

**Table III (Same as Table S1 in the manuscript) Data sets tested in the study**

| **datasets** | **No. cells** | **No. clusters** | **No. markers** |
| --- | --- | --- | --- |
| Levine13dim | 81,747 | 24 | **13** |
| Levine32dim | 104,184 | 14 | **32** |
| Samusik01 | 53,173 | 24 | **39** |
| muscle | 585,133 | 8 | **25** |
| Cell Cycle | 81,594 | 4 | **35** |
| colon | 130,667 | 13 | **19** |

**Figure III (Same as Fig. S2 in the manuscript)** Impact of limited training sets on the performance of LDA.

(3) The datasets used in this study contain cell population labels for around half of all

cells, with the remaining cells designated as "unassigned". In this study, the authors have excluded the unassigned cells -- so all methods are run on the labeled cells only (see Methods). This creates a much easier clustering (unsupervised) or classification (supervised) task, since the unassigned cells would be exactly the cells where the clustering or classification task is more difficult. This design choice should either be clearly justified in the text, or changed to include all cells (which would likely significantly affect performance for all methods).

A related concern is that it is possible that the "true" cluster labels may be incorrect for some cells in some datasets. This issue should be discussed in the text, in the context of both unsupervised and supervised methods.

*Response to Major comment 3: Thanks for the valuable suggestion on these important questions, which we did not discuss enough in the previous manuscript. We have made point-to-point responses in below:*

“In this study, the authors have excluded the unassigned cells -- so all methods are run on the labeled cells only (see Methods). This creates a much easier clustering (unsupervised) or classification (supervised) task, since the unassigned cells would be exactly the cells where the clustering or classification task is more difficult.”

*We agree that the “intermediate” or “ambiguous” cells in manual gating pose a critical problem for researchers and is one of the motivations to replace manual gating by clustering tools. Admittedly, discarding unlabeled cells may miss some useful information. As suggested by the reviewer, the decision and the related discussion has been clearly justified in METHODS sections (manuscript pages 24-25, lines 496-500). However, we believe it’s proper to not include the unlabeled cells from Levine13dim, Levine32dim and Samusik01 data into our analysis for two reasons:*

*A. According to the gating strategy of these data, most of the unlabeled cells were
assigned “unlabeled” since they were considered as unqualified cells and located at the gate of “cell length” or “DNA volume”, which made them not suitable for being clustered. Few of unlabeled cells were assigned “unlabeled” since they located at the intermediate gate. Although the few intermediate cells were worthy of clustering, we cannot distinguish them from the unlabeled cells. Therefore, we did not consider them to cluster. Maybe it is due to this fact that the comparison work of Weber and Robinson (Weber and Robinson 2016) also does not include the unlabeled cells into clustering.*

*B. The three new data sets we included in the revised manuscript were fully annotated, which in part overcome the problem. More specifically, muscle data contained a Neg/Neg population, which included all cells without known cell markers.*

“A related concern is that it is possible that the "true" cluster labels may be incorrect for some cells in some datasets.”

*The problem of the putative mistakes of true labels is also of importance in the current study. Two of our results are related to address this issue and have been discussed in the manuscript:*

*A. We found that the internal evaluation of semi-supervised tools was not as good as that of unsupervised tools, which suggests that semi-supervised tools did not ideally capture the inner structure of the CyTOF data (see manuscript page 11 and lines 211-217). This result supports the viewpoint that manual gating analyzes too few bi-axial plots and cannot fully depict the relation among features (Florian, 2016).*

*B. There exist quite a few biologically meaningful subtypes that are found by unsupervised tools but are not defined by manual gating (see manuscript pages 16-17 and lines 307-330). In fact, whether to define small subtypes or simply define major cell types is quite arbitrary in manual gating. Sometimes choosing to define rough clusters might blunt the difference among specific subtypes (Figure II).*

**Figure II-1 (Same as Fig. 4 in the manuscript)** Clustering resolution of Xshift and DEPECHE. A: Each row represents one cluster detected by Xshift; each column represents a manual label (left panel) of tissue origin (right panel). Color of each grid denotes the proportion of cells of that cluster belonging to the corresponding manual label (left) of tissue (right). Thus, row sums of both panels are 100%. Black grids highlight the specific patterns resolved by Xshift. B: Similar to A, but for the relationship between DEPECHE clusters (column) and manual labels of Samusik01 data (row).

(4) The analysis regarding the number of clusters (e.g., Table 3) does not discuss the effects of parameter choices: e.g., for some methods, the number of clusters can easily be controlled by adjusting parameters.

Similarly, the "true" number of clusters in the original datasets depends on the choices made by the authors of the datasets. By using a different clustering resolution (i.e., larger or smaller clusters), it would be possible to define different "true" numbers of clusters in these datasets.

*Response to Major comment 4: We appreciate the reviewer’s insightful suggestion on the relation between parameter settings and clustering resolution. In the revised manuscript, we have examined this problem through several aspects and discussed them in the manuscript:*

*1. We ran each tool with their default settings to minimize the influence of parameter settings on the number of clusters.*

*2. There are widely applied alternative settings for Xshift (Elbow Plot determination) and FlowSOM (automatically estimate the number of clusters), which significantly impact the clustering resolution, so we also ran these settings and analyzed their number of clusters (see manuscript page 15 and lines 288-302, manuscript pages 21-22 and lines 428-448).*

*3. Considering the arbitrariness in resolution of manual labels (as mentioned by reviewer and our previous response), we no longer compared the identified number of clusters to a “real” number. We compared within each tool to evaluate the stability of each tool (Figure IV), then compared with other tools to analyze the differences in resolution.*

**Figure IV (Same as Fig. 4 in the manuscript) Number of clusters detected by each tool.** A&B: number of clusters detected by default settings of Accense, DEPECHE, Xshift, PhenoGraph and flowMeans. Translucent points represent results in 20,000 cell subsampling tests. C&D: number of clusters (C) and precision (D) of highlighted tools and settings were impacted by sample size in the Levine32dim dataset. Dotted lines represent performance of alternative settings of different tools (Xshift: Elbow Plot Determination; FlowSOM: automatic estimation of number of clusters).

(5) Runtimes and computational efficiency are not discussed. For a comprehensive comparison of methods, this is crucial information that should be included.

*Response to Major comment 5: In the revised manuscript, we have recorded runtimes in all subsampling tests and presented them in Figure V (manuscript pages 9-10 and lines 166-179). This result has been taken into consideration when building the decision pipeline for selecting appropriate tool and has been discussed in the manuscript.*

**Figure V (same as Figure 1 in the manuscript)** Runtime and F-measure of each tool.

(6) Code is not provided. Ideally, a code repository (e.g., GitHub) containing code to reproduce all results and figures in the paper should be provided, to ensure reproducibility of the results.

*Response to Major comment 6: Thanks for the suggestion. We have now uploaded all processed data sets to flowrepository or cytobank and stored our MATLAB, Python, and R code on GitHub. The detailed information is recorded in the Availability of data and materials section (manuscript page 33 and lines 670-677).*

Minor comments

Additional minor comments:

* Background (lines 34-35): mentions that "CyTOF achieves a much higher throughput (up to millions of cells vs. thousands of cells)" compared to single-cell RNA-sequencing. This is not strictly true, since recent scRNA-seq studies have now also reached more than a million cells (e.g., Cao et al., 2019: <https://www.ncbi.nlm.nih.gov/pubmed/30787437>).

*Response to Minor comment 1: We thank the reviewer for pointing out this imprecise statement. This sentence has been corrected to state that the majority of single cell RNA data have the magnitude of tens to hundreds of thousands of cells (manuscript page 3 and lines 38-41).*

*	Background (line 36): mentions "millions of cell subtypes". There is no current
evidence that there are this many cell subtypes.

*Response to Minor comment 2: Thanks for the comment. This sentence has been re­worded to express that millions of cells are partitioned into subtypes (manuscript page 3 and line 41).*

* Background (line 77): mentions that the study by Weber and Robinson (2016) evaluated clustering accuracy but not stability. This is not correct: this study also evaluated stability (although the main focus was on clustering performance and runtimes).

*Response to Minor comment 3: We thank the reviewer for the comment. We have modified the corresponding sentences to express that previous studies mainly focus on clustering performance and runtimes, and their works can be integrated to give a more comprehensive guideline (manuscript page 5 and lines 83-85).*

*	Results (line 115): these values (for accuracy, F measure, and normalized mutual

information) do not appear to be shown in the corresponding table (Table 2).

*Response to Minor comment 4: Thanks for the comment on the result visualization. We have avoided such description and presented our result in Table 2 and Additional file 2 to make them more explicit.*

**Table IV(Same as Table 2 in the manuscript)**

| **Datasets** | **Methods** |  | **External evaluations** | |  |
| --- | --- | --- | --- | --- | --- |
|  |  | **Accuracy** | **F-measure** | **NMI** | **ARI** |
|  | Accense |  |  |  |  |
|  |  | 0.3529±0.0471 | 0.3500±0.0636 | 0.2490±0.0240 | 0.1682±0.0395 |
|  | PhenoGraph |  |  |  |  |
|  |  | 0.2309±0.0268 | 0.2789±0.0196 | 0.1364±0.0087 | 0.0683±0.0074 |
|  | Xshift |  |  |  |  |
|  | kmeans | 0.3622±0.0419 | 0.3970±0.0383 | 0.1710±0.0165 | 0.0752±0.0353 |
|  | flowMeans | 0.3969±0.0021 | 0.4224±0.0019 | 0.0963±0.0016 | 0.0681±0.0016 |
| Cell Cycle |  | 0.3055±0.0061 | 0.3506±0.0051 | 0.1849±0.0047 | 0.0694±0.0058 |
|  | FlowSOM |  |  |  |  |
|  |  | 0.6605±0.0021 | 0.6897±0.0029 | 0.1040±0.0035 | 0.1171±0.0025 |
|  | DEPECHE |  |  |  |  |
|  |  | 0.2808±0.0126 | 0.3551±0.0110 | 0.1361±0.0246 | 0.0546±0.0197 |
|  | ACDC |  |  |  |  |
|  |  | 0.8342±0.0071 | 0.8466±0.0093 | 0.4325±0.0212 | 0.5579±0.0129 |
|  | LDA |  |  |  |  |
|  |  | 0.9095±0.0006 | 0.9110±0.0005 | 0.6189±0.0032 | 0.7225±0.0021 |
|  | Accense |  |  |  |  |
|  |  | 0.3209±0.0353 | 0.3495±0.0442 | 0.4504±0.0286 | 0.2304±0.0304 |
| Colon |  |  |  |  |  |
|  | PhenoGraph |  |  |  |  |
|  |  | 0.3772±0.0201 | 0.3994±0.0137 | 0.4254±0.0073 | 0.2696±0.0094 |

| **Datasets** | **Methods** |  | **External evaluations** | |  |
| --- | --- | --- | --- | --- | --- |
|  |  | **Accuracy** | **F-measure** | **NMI** | **ARI** |
|  | Xshift |  |  |  |  |
|  | kmeans | 0.3187±0.0167 | 0.3094±0.0124 | 0.3763±0.0076 | 0.2399±0.0299 |
|  | flowMeans | 0.4480±0.0216 | 0.4951±0.0086 | 0.4688±0.0073 | 0.3235±0.0132 |
|  |  | 0.5095±0.0894 | 0.5901±0.0582 | 0.4705±0.0619 | 0.3724±0.1125 |
|  | FlowSOM |  |  |  |  |
|  |  | 0.5597±0.0284 | 0.5888±0.0230 | 0.5303±0.0157 | 0.4157±0.0288 |
|  | DEPECHE |  |  |  |  |
|  |  | 0.5902±0.0186 | 0.6898±0.0132 | 0.4694±0.0239 | 0.5042±0.0188 |
|  | ACDC |  |  |  |  |
|  |  | 0.7439±0.0026 | 0.7874±0.0076 | 0.5705±0.0088 | 0.5952±0.0041 |
|  | LDA |  |  |  |  |
|  |  | 0.8576±0.0011 | 0.8587±0.0012 | 0.7410±0.0012 | 0.7626±0.0017 |
|  | Accense |  |  |  |  |
|  |  | 0.6055±0.0946 | 0.6745±0.0929 | 0.7486±0.0603 | 0.6408±0.1034 |
|  | PhenoGraph |  |  |  |  |
|  |  | 0.8880±0.0015 | 0.9123±0.0167 | 0.8639±0.0078 | 0.8884±0.0159 |
|  | Xshift |  |  |  |  |
|  | kmeans | 0.7573±0.0091 | 0.7606±0.0125 | 0.7359±0.0118 | 0.7465±0.0116 |
|  | flowMeans | 0.5684±0.0504 | 0.6293±0.0325 | 0.7070±0.0193 | 0.5721±0.0465 |
| Levine13dim |  | 0.8470±0.0486 | 0.8842±0.0343 | 0.8352±0.0282 | 0.8349±0.0459 |
|  | FlowSOM |  |  |  |  |
|  |  | 0.8540±0.0235 | 0.8760±0.0200 | 0.8590±0.0097 | 0.8732±0.0168 |
|  | DEPECHE |  |  |  |  |
|  |  | 0.6929±0.0142 | 0.8010±0.0077 | 0.6687±0.0099 | 0.6571±0.0118 |
|  | ACDC |  |  |  |  |
|  |  | 0.9010±0.0029 | 0.9275±0.0026 | 0.8635±0.0041 | 0.9011±0.0052 |
|  | LDA |  |  |  |  |
|  |  | 0.9582±0.0005 | 0.9586±0.0005 | 0.9275±0.0008 | 0.9539±0.0007 |
|  | Accense |  |  |  |  |
|  |  | 0.5514±0.0794 | 0.6008±0.0627 | 0.6876±0.0389 | 0.5289±0.0740 |
|  | PhenoGraph |  |  |  |  |
|  |  | 0.6369±0.0253 | 0.7062±0.0213 | 0.7410±0.0142 | 0.6437±0.0240 |
|  | Xshift |  |  |  |  |
| Levine32dim | kmeans | 0.7543±0.0605 | 0.7706±0.0469 | 0.7690±0.0323 | 0.7593±0.0737 |
|  | flowMeans | 0.5753±0.0413 | 0.6748±0.0231 | 0.7302±0.0115 | 0.6405±0.0638 |
|  |  | 0.9216±0.0318 | 0.9279±0.0337 | 0.9115±0.0290 | 0.9397±0.0342 |

| **Datasets** | **Methods** |  | **External evaluations** | |  |
| --- | --- | --- | --- | --- | --- |
|  |  | **Accuracy** | **F-measure** | **NMI** | **ARI** |
|  | FlowSOM |  |  |  |  |
|  |  | 0.8787±0.0975 | 0.8979±0.0664 | 0.8840±0.0600 | 0.8803±0.1267 |
|  | DEPECHE |  |  |  |  |
|  |  | 0.8931±0.0010 | 0.9231±0.0009 | 0.8436±0.0008 | 0.9297±0.0009 |
|  | ACDC |  |  |  |  |
|  |  | 0.9943±0.0006 | 0.9939±0.0007 | 0.9380±0.0052 | 0.9791±0.0020 |
|  | LDA |  |  |  |  |
|  |  | 0.9809±0.0003 | 0.9807±0.0004 | 0.9595±0.0006 | 0.9830±0.0002 |
|  | Accense |  |  |  |  |
|  |  | 0.3687±0.0663 | 0.4072±0.0743 | 0.3953±0.0391 | 0.2397±0.0759 |
|  | PhenoGraph |  |  |  |  |
|  |  | 0.3930±0.0325 | 0.4336±0.0249 | 0.3912±0.0083 | 0.2694±0.0261 |
|  | Xshift |  |  |  |  |
|  | kmeans | 0.5119±0.0591 | 0.5133±0.0463 | 0.3882±0.0190 | 0.3572±0.0405 |
|  | flowMeans | 0.6113±0.0046 | 0.6207±0.0045 | 0.4958±0.0030 | 0.4243±0.0055 |
| Muscle |  | 0.7880±0.0193 | 0.7928±0.0089 | 0.5841±0.0145 | 0.6364±0.0347 |
|  | FlowSOM |  |  |  |  |
|  |  | 0.8210±0.0068 | 0.8286±0.0073 | 0.6470±0.0072 | 0.6688±0.0175 |
|  | DEPECHE |  |  |  |  |
|  |  | 0.8346±0.0018 | 0.8850±0.0019 | 0.5792±0.0015 | 0.7074±0.0035 |
|  | ACDC |  |  |  |  |
|  |  | 0.8787±0.0101 | 0.8784±0.0089 | 0.6750±0.0168 | 0.7593±0.0190 |
|  | LDA |  |  |  |  |
|  |  | 0.9240±0.0011 | 0.9238±0.0011 | 0.7606±0.0031 | 0.8295±0.0031 |
|  | Accense |  |  |  |  |
|  |  | 0.5868±0.0502 | 0.6376±0.0400 | 0.7165±0.0237 | 0.5574±0.0290 |
|  | PhenoGraph |  |  |  |  |
|  |  | 0.9260±0.0412 | 0.9249±0.0344 | 0.8979±0.0285 | 0.9250±0.0526 |
|  | Xshift |  |  |  |  |
|  | kmeans | 0.8909±0.0485 | 0.9091±0.0324 | 0.8742±0.0196 | 0.8781±0.0561 |
|  |  | 0.4837±0.0572 | 0.5535±0.0401 | 0.6437±0.0186 | 0.4655±0.0482 |
| Samusik01 | flowMeans |  |  |  |  |
|  |  | 0.9064±0.0163 | 0.9099±0.0163 | 0.8818±0.0137 | 0.9206±0.0045 |
|  | FlowSOM |  |  |  |  |
|  |  | 0.8386±0.0693 | 0.8417±0.0668 | 0.8185±0.0639 | 0.8561±0.0732 |
|  | DEPECHE |  |  |  |  |
|  |  | 0.8300±0.0047 | 0.8677±0.0047 | 0.7271±0.0050 | 0.8298±0.0068 |
|  | ACDC |  |  |  |  |
|  |  | 0.9682±0.0027 | 0.9731±0.0019 | 0.9347±0.0047 | 0.9616±0.0021 |

**External evaluations**

**Datasets	Methods**

**Accuracy	F-measure	NMI	ARI**

LDA

0.9757±0.0002	0.9759±0.0002	0.9482±0.0004	0.9735±0.0005

Data shown as mean**±**standard deviation

*	Discussion (line 257): this sentence could be interpreted to mean that Abdelaal et al. (2019) are the original authors of the LDA algorithm; this should be re-worded (since the original LDA algorithm has existed for decades).

*Response to Minor comment 5: We thank the reviewer for the suggestion on this expression. Since this paragraph of the Discussion section has been completely rewritten in the revised manuscript, this sentence is no longer in the updated manuscript.*

Reviewer #2: Liu et al. tried to perform a benchmark comparison study of clustering methods for mass cytometry data and offer a guideline for the selection of clustering methods. The intention is good. However, the investigation is not thorough enough, and therefore it can't be accepted in its current status. I have some comments as below:

Major:

It is a self-proof question that the semi-supervised methods will outperform un­supervised methods since you have more domain prior knowledge to assist clustering. It spoils the manuscript if you list this finding as your top conclusion.

*Response to Major comment 1: We thank the reviewer for the insightful comment that comparing semi- and unsupervised tools in terms of clustering precision is less valuable. In the revised manuscript, we have performed additional evaluations and discussed the comparison results from three aspects: precision (external evaluation), coherence (internal evaluation), and stability (see RESULTS, manuscript pages 6-17 and lines 100-343).*

*1. We agreed that it was unfair to compare unsupervised tools to semi-supervised tools in terms of precision, so the analysis of precision (external evaluation) was conducted separately for the two categories to see which tools outperformed other tools of its type (see manuscript pages 7-10, lines 128-179). We no longer highlighted the granted perception that semi-supervised tools are more precise than unsupervised one. Instead, the core idea of the current study is that each tool has its own characteristics and best fits a specific situation. Researchers should choose the proper tool according to their data characteristics and his/her research purpose. Specifically, unsupervised tools can work well to discover unknown clusters. We verified this claim by showing that Xshift and Phenograph could find sub-clusters with biological significance that were not defined by manual gating (see manuscript pages 16-17 and lines 306-343, Figure II-1 & Figure II-2).*

*2. Meanwhile, we also discussed that semi-supervised tool is more effective when reliable priori information is available from two points:*

*a)	If we used the internal evaluations to measure the performance of ACDC and LDA methods, we found that there are no significant improvements of the two semi-supervised methods over the unsupervised methods. This is because the calculation of internal evaluations has nothing to do with the external manual labels, and they just faithfully measure whether the identified clustering is of well*

*within-group similarity and between-group dissimilarity. On this occasion, manual gating labels are no longer regarded as reliable priori information (see manuscript page 11, lines 211-217).*

*b)	LDA depends on a reliable, homogeneous training set. If we selected training set from single cell line (Cell cycle data) or tissue (colon data), the performance of LDA has declining trend over the performance of training set coming from different cell lines or tissues (see manuscript pages 8-9 and lines 150-161, Figure III).*

*3. On the other hand, given that internal evaluation is defined on the basis of maximizing both the within-group similarity and between-group dissimilarity, and it simply measures the quality of the clustering result itself without taking external manual labels into account (thus we call it “internal”), we infer that these internal indicators are capable of comparing the two method categories in an unbiased manner. Thus, we compared the internal evaluations of all tools together to see which tool best captures the inner structure of CyTOF data (Table I & third section of RESULTS corresponding to manuscript pages 10-11, lines 181-219).*

*In summary, we integrated results from different aspects and generated a decision pipeline (Figure I) to choose the proper tool. We believe that this pipeline is more valuable than* *isolated conclusions on specific aspects.*

**Figure II-1 (Same as Fig. 4 in the manuscript)** Clustering resolution of Xshift and DEPECHE. A: Each row represents one cluster detected by Xshift; each column represents a manual label (left panel) of tissue origin (right panel). Color of each grid denotes the proportion of cells of that cluster belonging to the corresponding manual label (left) of tissue (right). Thus, row sums of both panels are 100%. Black grids highlight the specific patterns resolved by Xshift. B: Similar to A, but for the relationship between DEPECHE clusters (column) and

manual labels of Samusik01 data (row).

**Figure II-2 (Same as Fig. S5 in the manuscript)** Clustering resolution for PhenoGraph (colon data) and DEPECHE (Levine13dim data).

**Figure III (Same as Fig. S2 in the manuscript)** Impact of limited training sets on the performance of LDA.

**Figure I (Same as Fig. 5 in the manuscript).** Upper panel: relative advantages of each tool. Note, precision and coherence are not exclusive; some tools like FlowSOM and PhenoGraph are both precise and coherent. Position on the graph roughly represents relative relation. Lower panel: summary of characteristics of each tool and suggested decision pipeline to choose the right tool.

**Table I (Same as Table 3 in the manuscript)**

| **Datasets** | **Methods** |  | **Internal evaluations** |  |
| --- | --- | --- | --- | --- |
|  |  | **CH** | **DB** | **XB** |
|  | Accense |  |  |  |
|  |  | 3.2409±0.2069 | 2.4746±0.2563 | 0.6473±0.0750 |
|  | PhenoGraph |  |  |  |
|  |  | 3.4250±0.0350 | 2.5692±0.1503 | 0.7864±0.0392 |
|  | Xshift |  |  |  |
|  | kmeans | 3.5620±0.0443 | 1.7337±0.1618 | 0.9131±0.3066 |
|  | flowMeans | 3.9414±0.0011 | 1.5971±0.0013 | 0.7705±0.0165 |
| Cell Cycle |  | 3.5992±0.0283 | 1.6237±0.0341 | 0.7294±0.0393 |
|  | FlowSOM |  |  |  |
|  |  | 3.6294±0.0036 | 1.3578±0.0351 | 0.6669±0.0269 |
|  | DEPECHE |  |  |  |
|  |  | 3.8372±0.0283 | 1.7767±0.0322 | 0.7695±0.0177 |
|  | ACDC |  |  |  |
|  |  | 3.4645±0.0901 | 2.0773±0.3156 | 0.9395±0.0428 |
|  | LDA |  |  |  |
|  |  | 3.4581±0.0036 | 2.5080±0.0191 | 1.0021±0.0358 |
|  | Accense |  |  |  |
| Colon |  | 3.2688±0.0622 | 1.7064±0.0656 | 1.0427±0.0545 |
|  |  | **24** |  |  |

|  |  |  | **Internal evaluations** |  |
| --- | --- | --- | --- | --- |
| **Datasets** | **Methods** |  |  |  |
|  |  | **CH** | **DB** | **XB** |
|  |  |  |  |  |
|  | PhenoGraph |  |  |  |
|  |  | 3.4231±0.0194 | 1.9030±0.0245 | 1.3997±0.1199 |
|  | Xshift |  |  |  |
|  | kmeans | 3.0706±0.0179 | 2.3218±0.0595 | 1.3207±0.0511 |
|  | flowMeans | 3.5109±0.0079 | 1.9037±0.0501 | 1.4570±0.1055 |
|  |  | 3.4793±0.1068 | 1.5864±0.0421 | 1.6706±0.1958 |
|  | FlowSOM |  |  |  |
|  |  | 3.6435±0.0117 | 1.6583±0.0514 | 1.5910±0.1568 |
|  | DEPECHE |  |  |  |
|  |  | 3.9819±0.0375 | 1.7725±0.0250 | 1.6325±0.0930 |
|  | ACDC |  |  |  |
|  |  | 3.5898±0.0440 | 2.0607±0.1160 | 1.4239±0.0754 |
|  | LDA |  |  |  |
|  |  | 3.5100±0.0009 | 2.1773±0.0065 | 1.5564±0.0724 |
|  | Accense |  |  |  |
|  |  | 3.4230±0.0932 | 1.8832±0.1408 | 1.2321±0.0278 |
|  | PhenoGraph |  |  |  |
|  |  | 4.0739±0.0176 | 1.4645±0.0346 | 1.3972±0.1349 |
|  | Xshift |  |  |  |
|  | kmeans | 3.5106±0.0289 | 2.4284±0.0443 | 1.7868±0.0476 |
|  | flowMeans | 3.8508±0.0150 | 2.1550±0.0546 | 1.6213±0.1471 |
| Levine13dim |  | 4.0475±0.0194 | 1.5030±0.0849 | 1.4234±0.1182 |
|  | FlowSOM |  |  |  |
|  |  | 3.8486±0.0071 | 1.7564±0.0615 | 1.5043±0.1531 |
|  | DEPECHE |  |  |  |
|  |  | 4.2783±0.0174 | 1.1677±0.0342 | 1.3562±0.0392 |
|  | ACDC |  |  |  |
|  |  | 3.9638±0.0110 | 1.4916±0.0370 | 1.3109±0.0948 |
|  | LDA |  |  |  |
|  |  | 3.8288±0.0106 | 2.0046±0.0493 | 1.3828±0.1167 |
|  | Accense |  |  |  |
|  |  | 3.4621±0.0901 | 2.3414±0.0925 | 0.7891±0.0950 |
|  | PhenoGraph |  |  |  |
|  |  | 3.7401±0.0081 | 1.8293±0.0810 | 1.0009±0.0479 |
| Levine32dim |  |  |  |  |
|  | Xshift |  |  |  |
|  | kmeans | 3.6669±0.0102 | 2.2576±0.1324 | 0.8295±0.1382 |
|  |  | 3.8761±0.0166 | 2.0587±0.0386 | 0.9972±0.0441 |
|  |  | 25 |  |  |

|  |  |  | **Internal evaluations** |  |
| --- | --- | --- | --- | --- |
| **Datasets** | **Methods** |  |  |  |
|  |  | **CH** | **DB** | **XB** |
|  |  |  |  |  |
|  | flowMeans |  |  |  |
|  |  | 3.8546±0.0393 | 1.6975±0.2199 | 0.7985±0.0709 |
|  | FlowSOM |  |  |  |
|  |  | 3.8244±0.0285 | 1.5974±0.0863 | 0.8366±0.0792 |
|  | DEPECHE |  |  |  |
|  |  | 4.1480±0.0009 | 1.4727±0.0023 | 0.7575±0.0351 |
|  | ACDC |  |  |  |
|  |  | 3.6169±0.0046 | 1.3974±0.0049 | 0.7693±0.1310 |
|  | LDA |  |  |  |
|  |  | 3.8297±0.0007 | 1.7011±0.0099 | 0.7155±0.0139 |
|  | Accense |  |  |  |
|  |  | 3.2254±0.1688 | 2.3190±0.3178 | 0.8420±0.1211 |
|  | PhenoGraph |  |  |  |
|  |  | 3.6052±0.0132 | 1.8619±0.0417 | 1.7228±0.2389 |
|  | Xshift |  |  |  |
|  | kmeans | 3.2898±0.0274 | 2.2460±0.1524 | 2.1455±0.1599 |
|  | flowMeans | 3.9722±0.0022 | 1.7729±0.0099 | 1.4987±0.1212 |
| Muscle |  | 3.3809±0.0565 | 1.7685±0.0769 | 1.3750±0.1428 |
|  | FlowSOM |  |  |  |
|  |  | 3.8262±0.0146 | 1.4439±0.0462 | 1.3586±0.1473 |
|  | DEPECHE |  |  |  |
|  |  | 4.2639±0.0044 | 1.2235±0.0080 | 1.2892±0.0269 |
|  | ACDC |  |  |  |
|  |  | 3.6900±0.0305 | 1.7186±0.0515 | 1.5641±0.15555 |
|  | LDA |  |  |  |
|  |  | 3.8073±0.0031 | 1.6641±0.0206 | 1.6003±0.0900 |
|  | Accense |  |  |  |
|  |  | 3.2952±0.0476 | 2.3424±0.1454 | 0.6500±0.0266 |
|  | PhenoGraph |  |  |  |
|  |  | 3.7380±0.0033 | 1.4607±0.0424 | 0.7669±0.0831 |
|  | Xshift |  |  |  |
|  | kmeans | 3.2660±0.0134 | 2.7855±0.4644 | 1.2670±0.1654 |
| Samusik01 | flowMeans | 3.7087±0.0135 | 2.2072±0.0854 | 0.9365±0.0792 |
|  |  | 3.4029±0.0381 | 1.6069±0.2374 | 0.6309±0.0475 |
|  | FlowSOM |  |  |  |
|  |  | 3.6941±0.0257 | 1.8967±0.3799 | 0.8286±0.0832 |
|  | DEPECHE |  |  |  |
|  |  | 4.1028±0.0021 | 1.4867±0.0054 | 0.9141±0.0109 |
|  |  | **26** |  |  |

**Internal evaluations**

**Datasets	Methods**

**CH	DB	XB**

ACDC

3.6827±0.0178	1.3871±0.0132	0.7273±0.0573

LDA

3.6767±0.0080	1.6325±0.0242	1.0414±0.0808

Data shown as mean ± standard deviation. CH: Calinski-Harabasz index (log10 transformed), DB: Davies-Bouldin index, XB: Xie-Beni index (log10 transformed).

There are so many clustering methods out there. The authors compared too few methods.

*Response to Major comment 2: We thank the reviewer for the comment on the selection of tools and data sets. We have expanded our inclusion of both examined methods and data sets. In the current version, a total of nine tools were included: Accense, DEPECHE, FlowSOM, flowMeans, kmeans, PhenoGraph, Xshift, ACDC, and LDA (see METHODS in the first section of Clustering tools, Table I, manuscript page 23 and lines 459-476). Among them, PhenoGraph, Xshift, flowSOM, flowMeans were the best-performing tools in a previous comparison by Weber and Robinson (2016), DEPECHE is a novel unsupervised clustering tools for cytometry data that was not previously analyzed by their benchmark, and LDA and ACDC were top-performing tools in a previous comparison of semi-supervised tools by Abdelaal et al. We believe that the previous benchmarking comparisons by Weber & Robinson and Abdelaal et al. have highlighted the strength of each tool of its kind (semi- or unsupervised) and narrowed the range of tools to choose from. From these benchmarks, we now have a great opportunity to give a focused and detailed comparison to find the best fit situation for each of these popular tools. In addition, two moderate tools, kmeans and Accense (density-based), were included to analyze the potential bias between density-based and kmeans-based tools. One of our innovations in this study is that we not only analyze the coherence as internal evaluations (CH, DB and XB) to capture the inner structure of dataset, but also explore the possible relation between internal evaluation indicators and the basic principle of methods. For example, CH and DB naturally favor kmeans-based methods while XB favor density-based methods. However, these facts were not discussed in the previous publications.*

*In addition, we also expanded three additional novel data sets in our comparison: muscle, Cell Cycle, and colon (METHODS, in the section of Datasets, Table III, manuscript page 24 and lines 478-494). We believe that the inclusion of distinct, heterogeneous data sets can more comprehensively evaluate the performance of each tool in different situations.*

*1. Muscle-resident cells from normal adult mice (Giordani et al., 2019). 25 proteins were
used for clustering. 8 major cell populations were identified following provided gating strategy, including Neg/Neg cells that lacked any known cell markers (manuscript lines 484-486).*

*2. Cell Cycle data from in vitro cells of three cell lines: HEK293T, MDA-MB-231, and THP-1 (Rapsomaniki et al., 2018). These cell lines were treated by TNFα to induce a cell cycle transformation. Cells at different time points after treatment were collected. Cells were labeled by four phases: G0/G1, G2, S and M. A total of 35 markers were measured (manuscript lines 487-490).*

*3. Our laboratory’s human colon data: cells from healthy colon tissue, polyp of healthy adult, early stage colon cancer, and late stage colon cancer. There were 19 protein markers and 13 manual labels (Zhang et al., 2019, manuscript lines 491-494).*

*Compared to the previously included data sets, these new data have some distinct characteristics that are useful for testing the performance of clustering tools:*

*1. They have relatively small numbers of manual labels, which helps us better evaluate the relation between the arbitrariness of manual gating and the performance of unsupervised tools. An extreme example is Cell Cycle data, which has only four labels.*

*2. They come from solid tissue (muscle), solid tumors (colon cancer), and in vitro cell curations (Cell Cycle). Together with previous data sets that come from bone marrow, our comparison roughly reflects the performance of each tool on different tissue types.*

*3. Cell Cycle and colon cancer data contain heterogenous samples (cells from different cell lines or tissue types). As shown by our results (Figure III), the heterogeneity of data significantly influenced the performance of several tools. Therefore, the robustness of a tool against the heterogeneity of the original sample is another comparison in our analysis, which has seldom been mentioned in previous literature.*

**Table II (Same as Table 1 in the manuscript) Methods compared in the study.**

| **Methods** | | **Implemen  tation  tools** | **Description** | **ref** |
| --- | --- | --- | --- | --- |
| **unsupervise d** | Accense | MATLAB | tSNE dimension reduction & 2D projection  Kernel-based estimation of density  Density-based peak-finding and partitioning | [12] |
|  | PhenoGraph | R (cytofkit package) | Detection of k-nearest neighbors of each cell  Jaccard similarity coefficient as connectivity  Community detection based on connection density | [15] |
|  | Xshift | Vortex | Weighted k-nearest neighbor density estimation  Detection of density centroids  Cells linked to centroid via density-ascending paths | [18] |
|  | FlowSOM | R | Self-organizing map (SOM) trained on scaled data  Nodes of SOM connected by minimal spanning  tree  Consensus hierarchical meta-clustering of nodes | [37] |
|  | flowMeans | R | K estimated by peak numbers of kernel density  kmeans clustering of estimated K  Merging clusters by distance metrics | [26] |
|  | DEPECHE | R | Tuning penalty by resampling data set  Penalized kmeans clustering | [32] |
|  | kmeans | MATLAB | Standard kmeans procedure |  |
| **semi-supervised** | ACDC | Python | Marker×cell matrix & cell type×marker table  Detect landmark points by community detection | [10] |

Link cells to landmarks by random walkers

LDA	MATLAB	Linear discriminant analysis with training data sets	[8]

**Table III (Same as Table S1 in the manuscript) Data sets tested in the study**

| **datasets** | **No. cells** | **No. clusters** | **No. markers** |
| --- | --- | --- | --- |
| Levine13dim | 81,747 | 24 | **13** |
| Levine32dim | 104,184 | 14 | **32** |
| Samusik01 | 53,173 | 24 | **39** |
| muscle | 585,133 | 8 | **25** |
| Cell Cycle | 81,594 | 4 | **35** |
| colon | 130,667 | 13 | **19** |

**Figure III (Same as Fig. S2 in the manuscript)** Impact of limited training sets on the performance of LDA.

The review of the methods is not very precise. For example, what does "manual curated knowledge" exactly refer to?

*Response to Major comment 3: We apologize for the confusion. This expression has been replaced by “predetermined manual labels”. We also rewrote some of the ambiguous sentences in BACKGROUND (manuscript page 4 and line 60).*

About clustering result measurement: Page 18, please specify what is precision and recall. Are they related with map(Ti)? There is a straightforward metric to measure the similarity of clustering results called adjusted rand index. The authors can consider using it too.

*Response to Major comment 4: We thank the reviewer for the suggestion on evaluation metrics.*

“please specify what is precision and recall.”

*We’ve specified the definition of precision and recall as follow (METHODS, manuscript pages 28 and lines 575-579):*

$$P_{i}=\frac{true positive}{true positive+false positive}$$

$$R_{i}=\frac{true positive}{true positive+false negative}$$

“Are they related with map(Ti)?”

*Map (Ti) is a one-to-one mapping function for matching predicted labels and true labels (METHODS, manuscript page 28 and lines 568-570). It is well known that the labels obtained by most clustering methods do not match with the predefined true labels one by one. However, the calculation of some evaluation metrics, such as accuracy and F-measure (precision, recall), first require that two types of labels match, so we adopt a map (Ti) of the Hungarian Maximum Matching Algorithm to implement in our study.*

There is a straightforward metric to measure the similarity of clustering results called adjusted rand index. The authors can consider using it too.

*In the revised manuscript, the adjusted rand index (ARI) was also included as an external evaluation. Results of ARI were in agreement with F-measure and NMI, which further verified our results (METHODS, manuscript pages 29-30 and lines 599-604).*

Did the authors consider comparing the running time of these methods?

*Response to Major comment 4: Thanks for the suggestion. In the revised manuscript, we have recorded runtimes in all subsampling tests and presented them in Figure V (manuscript pages 9-10 and lines 166-179). This result has been taken into consideration when building the decision pipeline and has been discussed in the manuscript.*

**Figure V (same as Figure 1 in the manuscript)** Runtime and F-measure of each tool.

Minor:

Page 3, line 36, "millions of cell subtypes", is this a reasonable magnitude?

*Response to Minor comment 1: We apologize for the misunderstanding. This sentence*

*has been re-worded to express that millions of cells are partitioned into subtypes (manuscript page 3 and lines 38-41).*

Grammar mistakes, e.g., absent VS. absence, hypothesis VS. hypothesize. Please check thoroughly.

*Response to Minor comment 2: Thanks for the comments. We have updated our manuscript thoroughly to minimize the occurrence of grammar mistakes. We have employed an English native speaker to proofread and correct the grammar mistakes throughout the manuscript.*

**Second round of review**

**Reviewer 1**

Overall comments

In this resubmission, Liu et al. have expanded the number of unsupervised methods included in the comparison, and included additional datasets and performance metrics. However, the central concern of comparing supervised and unsupervised methods within the same framework has not been sufficiently addressed. Supervised and unsupervised methods perform conceptually distinct analysis tasks, so comparing these within a single study - using some of the same datasets and metrics - does not make sense conceptually, and provides confusing information to readers. In addition, the expanded set of methods still largely includes only methods from previous comparisons (only one new method is included - DEPECHE).


Major comments

1) As discussed in the previous set of comments, the study compares supervised classification methods and unsupervised methods (i.e. clustering methods) within the same framework, using some of the same evaluation metrics (e.g., F-measure). For example, Figure 1 shows F-measures for both supervised and unsupervised tools on the same figures. However, this is not a fair comparison, and does not make sense conceptually - supervised tools make use of extra information in the form of the ground truth class labels, while unsupervised tools ignore any class labels.

Similarly, the last paragraph of the abstract (and last paragraph of the introduction, line 97) suggest to readers that the choice between supervised and unsupervised methods comes down to performance metrics such as precision, stability, etc. Again, this reflects a misunderstanding of the role of supervised and unsupervised methods - in fact, the choice will be determined by the experimental setup, i.e., whether ground truth class labels are available (supervised) or not (unsupervised).

This is also reflected in the use of terminology - it does not really make sense to refer to supervised methods as "clustering" tools.


2) Similarly, one of the major results presented from the new performance metrics (coherence / internal evaluation; line 371 onwards) is that clusters generated by unsupervised tools have higher internal coherence than populations recovered by the supervised tools. Again, this comparison is confusing, since these two classes of tools are performing different tasks - unsupervised tools will by definition tend to generate coherent clusters, while supervised tools assign cells to the available ground truth categories.


3) The authors have expanded the set of methods included, and included additional datasets and performance metrics. The set of methods now includes the top-performing unsupervised methods from previous studies (e.g., FlowSOM). However, overall, the study still does not significantly expand on the sets of methods from previous comparisons - only one new unsupervised method (DEPECHE) compared to the earlier study by Weber and Robinson (2016); and no new supervised methods compared to the study by Abdelaal et al. (2019).

Therefore, while the revised study provides new information on the performance of tools that have previously been compared, it largely (apart from one new unsupervised method) does not provide any fundamentally new information for readers.


4) The authors have responded to point (3) from the previous comments that, in their view, it is justified to exclude "unassigned" cells (i.e., cells with no ground truth cluster labels) when running the unsupervised (clustering) algorithms, and that previous studies have also done this. This is incorrect - the previous study by Weber and Robinson (2016) included all cells when running the clustering algorithms, and then calculated performance measures using the subset of labeled cells.

By excluding "unassigned" cells prior to clustering (also mentioned on lines 496-498 in Methods), the authors have effectively created a much easier clustering task for the algorithms. In practice, when readers run clustering algorithms on new datasets, they will not have access to any ground truth labels, so they will need to run the algorithms on all cells. Therefore, this choice of methodology significantly reduces the usefulness of the results for readers, and needs to be clearly explained and justified in the text (i.e., clustering methods will be expected to perform more poorly on new datasets).

**Reviewer 2**

On response to the reviewer's comments, the authors revised the article one-by-one, the following main comments have been addressed during the revision.

1, it was proposed by both reviewer 1 and 2 that using the same metrics such as F measure to compare the performance of unsupervised and supervised methods is not meaningful, because the labeling knowledge will help supervised method to work better. In the revised version, the authors compared the performance of unsupervised and supervised methods separately, and they also used the internal evaluations to check which method can get the best inner structure of the data. In addition, a comprehensive description of each method was added, which will assist users to select the optimal method according to their dataset character.

2, The authors added 3 additional unsupervised methods to the original 6 methods, and the number of benchmark datasets increased to 6 by adding 3 types of new datasets. The increasement of both methods and datasets will make the comparison more comprehensive.

3, To the issue of "unassigned" cell exclusion and possible incorrect cluster labels in the datasets, the authors added reasonable explanations that the "unassigned" cells are considered unqualified cells and could not be clustered using the gating strategy. For the cell cluster labeling problem, the authors addressed this issue using two results in the text.

4, To issue the relation between the number of clusters and method parameter adjustment, the authors found that for each clustering method and dataset, the parameter setting can affect the number of clusters and the cluster resolutions.

5, the authors added the runtimes and computational efficiency comparison between varied methods which will help users to select appropriate method.

6, the users uploaded their code into Github.

7, other tiny comments were changed by the authors.

In summary, the authors correspondingly revised the article according to the reviewer's comments and the revised version is acceptable.

**Authors Response**

**Point-by-point responses to the reviewers’ comments:**

*Response to reviewer 1 comment 1:*

*1. As stated in the previous response letter, we agree with the reviewer 1 that external evaluations like F measure should be applied separately for supervised and unsupervised method, and that’s what we exactly did in the revised manuscript. In fact, external evaluations for supervised and unsupervised methods were conducted in two separate sections (line 128 onwards and line 163 onwards). This is also acknowledged by the reviewer 2. Maybe the arrangement of the table, figure and abstract confuses reviewer 1, and we would be glad to renew them to avoid the misunderstanding like “supervised methods should be chosen because they are precise”.*

*2. With regards to the “ground truth”, the first thing we must point out is that, unlike other fields of clustering analysis, the prior knowledge of CyTOF analysis is actually the binary relation between labels and features rather than the manual label itself (Spitzer, Cell 2016). Such prior knowledge can only partially capture the refined inner structure of the data; with more prior knowledge one may be able to capture more refined structure, but seldom can we achieve a perfect structure (we conducted an additional analysis on this topic, Table 1, which found that coherence of manual labels is usually lower than that of unsupervised clustering results, see Table 2).*

**Table 1 (the same as additional file Table S3) Internal evaluation for manual labels**

|  | Calinski-Harabasz | Davies-Bouldin | Xie-Beni |
| --- | --- | --- | --- |
| Levine13dim | 3.815192 | 2.401535 | 1.383491 |
| Levine32dim | 3.8251618 | 1.7089997 | 0.7910288 |
| Samusik01 | 3.6605007 | 1.7133195 | 0.7552018 |
| muscle | 3.755963 | 1.746056 | 1.538259 |
| Cell Cycle | 3.446701 | 2.511195 | 1.049529 |
| colon | 3.471482 | 2.930353 | inf |

**Table 2(the same as Table 3 in manuscript) Summary of internal evaluations**

| **Datasets** | **Methods** |  | **Internal evaluations** |  |
| --- | --- | --- | --- | --- |
|  |  | **CH** | **DB** | **XB** |
|  | Accense |  |  |  |
|  |  | 3.2409±0.2069 | 2.4746±0.2563 | 0.6473±0.0750 |
|  | PhenoGraph |  |  |  |
|  |  | 3.4250±0.0350 | 2.5692±0.1503 | 0.7864±0.0392 |
|  | Xshift |  |  |  |
|  | kmeans | 3.5620±0.0443 | 1.7337±0.1618 | 0.9131±0.3066 |
|  | flowMeans | 3.9414±0.0011 | 1.5971±0.0013 | 0.7705±0.0165 |
| Cell Cycle |  | 3.5992±0.0283 | 1.6237±0.0341 | 0.7294±0.0393 |
|  | FlowSOM |  |  |  |
|  |  | 3.6294±0.0036 | 1.3578±0.0351 | 0.6669±0.0269 |
|  | DEPECHE |  |  |  |
|  |  | 3.8372±0.0283 | 1.7767±0.0322 | 0.7695±0.0177 |
|  | ACDC |  |  |  |
|  |  | 3.4645±0.0901 | 2.0773±0.3156 | 0.9395±0.0428 |
|  | LDA |  |  |  |
|  |  | 3.4581±0.0036 | 2.5080±0.0191 | 1.0021±0.0358 |
|  | Accense |  |  |  |
|  |  | 3.2688±0.0622 | 1.7064±0.0656 | 1.0427±0.0545 |
|  | PhenoGraph |  |  |  |
|  |  | 3.4231±0.0194 | 1.9030±0.0245 | 1.3997±0.1199 |
|  | Xshift |  |  |  |
|  | kmeans | 3.0706±0.0179 | 2.3218±0.0595 | 1.3207±0.0511 |
|  | flowMeans | 3.5109±0.0079 | 1.9037±0.0501 | 1.4570±0.1055 |
| Colon |  | 3.4793±0.1068 | 1.5864±0.0421 | 1.6706±0.1958 |
|  | FlowSOM |  |  |  |
|  |  | 3.6435±0.0117 | 1.6583±0.0514 | 1.5910±0.1568 |
|  | DEPECHE |  |  |  |
|  |  | 3.9819±0.0375 | 1.7725±0.0250 | 1.6325±0.0930 |
|  | ACDC |  |  |  |
|  |  | 3.5898±0.0440 | 2.0607±0.1160 | 1.4239±0.0754 |
|  | LDA |  |  |  |
|  |  | 3.5100±0.0009 | 2.1773±0.0065 | 1.5564±0.0724 |
|  | Accense |  |  |  |
|  |  | 3.4230±0.0932 | 1.8832±0.1408 | 1.2321±0.0278 |
|  | PhenoGraph |  |  |  |
| Levine13dim |  | 4.0739±0.0176 | 1.4645±0.0346 | 1.3972±0.1349 |
|  | Xshift |  |  |  |
|  |  | 3.5106±0.0289 | 2.4284±0.0443 | 1.7868±0.0476 |

|  |  |  | **Internal evaluations** |  |
| --- | --- | --- | --- | --- |
| **Datasets** | **Methods** |  |  |  |
|  |  | **CH** | **DB** | **XB** |
|  |  |  |  |  |
|  | kmeans |  |  |  |
|  | flowMeans | 3.8508±0.0150 | 2.1550±0.0546 | 1.6213±0.1471 |
|  |  | 4.0475±0.0194 | 1.5030±0.0849 | 1.4234±0.1182 |
|  | FlowSOM |  |  |  |
|  |  | 3.8486±0.0071 | 1.7564±0.0615 | 1.5043±0.1531 |
|  | DEPECHE |  |  |  |
|  |  | 4.2783±0.0174 | 1.1677±0.0342 | 1.3562±0.0392 |
|  | ACDC |  |  |  |
|  |  | 3.9638±0.0110 | 1.4916±0.0370 | 1.3109±0.0948 |
|  | LDA |  |  |  |
|  |  | 3.8288±0.0106 | 2.0046±0.0493 | 1.3828±0.1167 |
|  | Accense |  |  |  |
|  |  | 3.4621±0.0901 | 2.3414±0.0925 | 0.7891±0.0950 |
|  | PhenoGraph |  |  |  |
|  |  | 3.7401±0.0081 | 1.8293±0.0810 | 1.0009±0.0479 |
|  | Xshift |  |  |  |
|  | kmeans | 3.6669±0.0102 | 2.2576±0.1324 | 0.8295±0.1382 |
|  | flowMeans | 3.8761±0.0166 | 2.0587±0.0386 | 0.9972±0.0441 |
| Levine32dim |  | 3.8546±0.0393 | 1.6975±0.2199 | 0.7985±0.0709 |
|  | FlowSOM |  |  |  |
|  |  | 3.8244±0.0285 | 1.5974±0.0863 | 0.8366±0.0792 |
|  | DEPECHE |  |  |  |
|  |  | 4.1480±0.0009 | 1.4727±0.0023 | 0.7575±0.0351 |
|  | ACDC |  |  |  |
|  |  | 3.6169±0.0046 | 1.3974±0.0049 | 0.7693±0.1310 |
|  | LDA |  |  |  |
|  |  | 3.8297±0.0007 | 1.7011±0.0099 | 0.7155±0.0139 |
|  | Accense |  |  |  |
|  |  | 3.2254±0.1688 | 2.3190±0.3178 | 0.8420±0.1211 |
|  | PhenoGraph |  |  |  |
|  |  | 3.6052±0.0132 | 1.8619±0.0417 | 1.7228±0.2389 |
|  | Xshift |  |  |  |
|  |  | 3.2898±0.0274 | 2.2460±0.1524 | 2.1455±0.1599 |
| Muscle | kmeans |  |  |  |
|  |  | 3.9722±0.0022 | 1.7729±0.0099 | 1.4987±0.1212 |
|  | flowMeans |  |  |  |
|  |  | 3.3809±0.0565 | 1.7685±0.0769 | 1.3750±0.1428 |
|  | FlowSOM |  |  |  |
|  |  | 3.8262±0.0146 | 1.4439±0.0462 | 1.3586±0.1473 |

|  |  |  | **Internal evaluations** |  |
| --- | --- | --- | --- | --- |
| **Datasets** | **Methods** |  |  |  |
|  |  | **CH** | **DB** | **XB** |
|  |  |  |  |  |
|  | DEPECHE |  |  |  |
|  |  | 4.2639±0.0044 | 1.2235±0.0080 | 1.2892±0.0269 |
|  | ACDC |  |  |  |
|  |  | 3.6900±0.0305 | 1.7186±0.0515 | 1.5641±0.15555 |
|  | LDA |  |  |  |
|  |  | 3.8073±0.0031 | 1.6641±0.0206 | 1.6003±0.0900 |
|  | Accense |  |  |  |
|  |  | 3.2952±0.0476 | 2.3424±0.1454 | 0.6500±0.0266 |
|  | PhenoGraph |  |  |  |
|  |  | 3.7380±0.0033 | 1.4607±0.0424 | 0.7669±0.0831 |
|  | Xshift |  |  |  |
|  | kmeans | 3.2660±0.0134 | 2.7855±0.4644 | 1.2670±0.1654 |
|  | flowMeans | 3.7087±0.0135 | 2.2072±0.0854 | 0.9365±0.0792 |
| Samusik01 |  | 3.4029±0.0381 | 1.6069±0.2374 | 0.6309±0.0475 |
|  | FlowSOM |  |  |  |
|  |  | 3.6941±0.0257 | 1.8967±0.3799 | 0.8286±0.0832 |
|  | DEPECHE |  |  |  |
|  |  | 4.1028±0.0021 | 1.4867±0.0054 | 0.9141±0.0109 |
|  | ACDC |  |  |  |
|  |  | 3.6827±0.0178 | 1.3871±0.0132 | 0.7273±0.0573 |
|  | LDA |  |  |  |
|  |  | 3.6767±0.0080 | 1.6325±0.0242 | 1.0414±0.0808 |

Data shown as mean ± standard deviation. CH: Calinski-Harabasz index (log10 transformed), DB: Davies-Bouldin index, XB: Xie-Beni index (log10 transformed).

*3. Another important fact is that such prior knowledge (i.e. the binary relation between labels and features) is almost always available. Reviewer 1 stated that the choice of supervised/unsupervised methods depends on experiment setup (i.e. is ground truth available), but this is imprecise: if the researchers do not use prior knowledge, they will find it hard to carry out downstream analysis for unsupervised clustering. This is because that the first step to interpret the clusters is often to give them labels according to the prior*

*knowledge of cell type-marker relation (Stuart, Nat Rev Genet 2019). Conversely, as long as these relations are available as prior knowledge, one can always generate manual labels by bi-axial gating (see point 4 below).*

*4. Consequently, we emphasize that in the field of CyTOF analysis, the manual label is not exactly the absolute “ground truth” like other fields (e.g. label “cancer/normal tissue” in deep learning of medical imagination); rather, it should be regarded as a clustering result derived from a simple and rigid clustering strategy (bi-axial plot gating) and carries some kinds of variation (Figure a).*

**Figure a: Uncertainty commonly exists during manual gating**

*Although researchers may practically accept manual labels as “golden standard”, it is in fact a special clustering result with some kinds of artificial bias. An astonishing fact by the Human Immune Genome Project is that, manual gating is one of the largest variables in the outcome of a flow cytometry-based experiment (Maecker, Nat Rev Immunol. 2012).*

*Another relating fact is that, although reviewer 1 questioned our nomenclature of “supervised cluster analysis”, this concept is actually accepted by the field (Grira, Nizar and Michel Crucianu. “Unsupervised and Semi-supervised Clustering : a Brief Survey ∗.” (2004). 314 citations on Google scholar).*

*5. We now go back to the original question of the choice between supervised and unsupervised methods. We would like to challenge the reviewer 1’s claim that the choice depends on experiment setup (i.e. is ground truth available?); instead, we suggest that it depends on the purpose of the experiment (i.e. do we care about known cell types only?) (Figure 5 Q1 in manuscript). Manual label has its pros and cons: on the one hand, it precisely captures the information we input in priori; on the other hand, it misses unknown information. We suggest that even if the manual label is always available, the choice of methods depends on how researchers evaluate the pros and cons according to his/her purpose of the study.*

*This conclusion is not self-evident; instead, it emerges from our results in this study. In Cell Cycle data, the manual knowledge of cell cycle captured very little proportion of the information, and different methods gave very different performance. If researchers care only about the known labels of cell cycle, his/her only choice is the supervised methods. However, if researchers wish to include other factors such as time and cell line into the cell clusters, unsupervised methods are more suitable. On the contrary, in Samusik01 data, prior knowledge is quite refined, and both methods gave comparable results. In both examples, prior knowledge (as well as manual label) is available, but the decision is very different.*

*Response to reviewer 1 comment 2:*

*(Relevant to point 4 in previous section) When we consider manual labels as a special form of clustering result, our efforts that compared the internal evaluations of both supervised and unsupervised clustering methods together would be reasonable. As mentioned above, the manual label of CyTOF data is obtained from manual clustering analysis, and its refinement and bias vary greatly. When manual labels as well as prior knowledge are highly refined, it could give good internal evaluation (coherent result). With this view, our results could be summarized as figure b (note, the number is not true and simply reflects the trend):*

**Figure b: an illustration to address comment 2 of Reviewer 1.**

*Of note, ACDC gave competitive internal performance in quite a few data sets, albeit not the best one. This is not surprising since ACDC utilizes prior knowledge to depict the characteristics of expression data, thus being able to partially capture the inner structure of data. Reviewer 1 questioned that supervised methods naturally have worse performance on internal evaluation. Given the above facts, we argue that even though our result is as expected, but definitely not trivial or self-evident. We would also like to point out that the result of “unsupervised methods have better internal evaluation than supervised methods” is indeed a small part of our internal evaluation, since many unsupervised methods were actually worse than ACDC. In fact, the key of this section is* *that we found distinct best-performance unsupervised methods (PhenoGraph and FlowSOM) for different conditions.*

*Response to reviewer 1 comment 3:*

*We acknowledge that the previous comparison work by Weber* [*et.al*](http://et.al) *have provided enriched information, and we agree with the view of Reviewer 1 that our study does not expand new methods from Weber* [*et.al*](http://et.al) *except one new unsupervised method (DEPECHE). For one thing, very few novel methods were recently developed special for mass cytometry data; furthermore, we find that most researchers remain use these classical methods to analyze their mass cytometry data(Gonzalez et al., 2018; Ajami et al., 2018). Therefore, our study takes more attention on the depth analyses of the most prevalent methods. For example, we discussed the influence of the varying sampling size of data on each method from two aspects: the robustness on precision and clustering resolution. We also explored the different performances of some methods with default and non-default settings. To the best of our knowledge, these aspects have been not discussed anywhere before, and this is the first attempt to provide a clear guideline of choosing clustering methods for mass cytometry data (Figure c).*

*To be specific, by taking varies factors like purpose of the study, sampling size, desired resolution of clustering and estimation of cluster number into account, we generated a clear decision guideline (Figure c) for beginners, which is valuable and not achieved by previous studies. Based on the above statement, we believe our study offer unique valuable information, just as the Reviewer 2’s comments that believe our work represent a significant advance over previously published studies (Figure d).*

**Figure c (the same as Figure 5 in manuscript): decision pipeline.**

**Figure d: the Reviewer 2’s comments consider a significant advance over previously published studies**

*Response to reviewer 1 comment 4:*

*First of all, it is worth noting that the ground true label in our manuscript is not the strictly truth, but a generally accepted clustering result by manual gating strategy, which means that each mass cytometry data can easily get such manual gating labels (i.e. “ground true” labels).*

*Through detailed discussion, we agree with the view of Reviewer 1 that the clustering methods shall run on all cells but calculate performance measures using the subset of labeled cells in the previous study by Weber and Robinson (2016). On this issue, we look forward to the opportunity to add this analysis to the current version of our manuscript. In addition, we believe that our study of discarding “unassigned” cells may also be reasonable for two reasons:*

*1) Most of the “unassigned” cells are usually low quality cells, intermediate cells or cells that do not express on some known markers, which means that these cells do not pass quality filtering, thus we believe it’s proper not to include them into the clustering. For example, by checking the gating strategies on Cytobank, we find that the majority of “unassigned” cells for Levine13dim and Levine32dim datasets were low quality and intermediate cells, and for Cell Cycle data were low quality cells. While for muscle data, the “unassigned” cells contain all the above three classes of cells. Given that the public Cell Cycle data obtains its manual gating labels by discarding some un-gated cells, and in order to compare each method in a unified way, we discarded “unassigned” cells for all datasets. Moreover, we can see that the number of analyzed cells are just equal to the number of cells discarding “unassigned” label in the publication of Ahmed Mahfouz et al. 2019, which further favor our view (Figure e).*

*2) Considering that each mass cytometry dataset can easily obtain its manual gating labels (i.e. “ground true” labels), although our study is performed on cells discarding some “unassigned” label, we believe it could be still applied on any new datasets and got desired results.*

**Figure e: the data description of Ahmed Mahfouz et al. 2019 aligns with our argument**
